# Correction: Repeated Exposure of Adult Rats to Transient Oxidative Stress Induces Various Long-Lasting Alterations in Cognitive and Behavioral Functions

**DOI:** 10.1371/journal.pone.0132171

**Published:** 2015-06-29

**Authors:** The PLOS ONE Staff

## Notice of Republication

This article was republished on June 11, 2015, to correct the Results section, in which degrees of freedom were mistakenly hyperlinked to references. The publisher apologizes for the error. S1 and S2 Tables were also corrected to remove tracked changes. Please download this article again to view the correct version. The originally published, uncorrected article and the republished, corrected article are provided here for reference.

## Supporting Information

S1 File Originally published, uncorrected article. (PDF)

S2 File Republished, corrected article. (PDF)

## References

[1] Iguchi Y, Kosugi S, Nishikawa H, Lin Z, Minabe Y, Toda S (2014) Repeated Exposure of Adult Rats to Transient Oxidative Stress Induces Various Long-Lasting Alterations in Cognitive and Behavioral Functions. PLoS ONE 9(12): e114024 doi:10.1371/journal.pone.0114024 25489939 10.1371/journal.pone.0114024 PMC4260961

