# Supplementary figures and images for: Repeated Exposure of Adult Rats to Transient Oxidative Stress Induces Various Long-Lasting Alterations in Cognitive and Behavioral Functions
Source: PLoS One. 2014 Dec 9;9(12):e114024. doi: 10.1371/journal.pone.0114024 (PMC4260961; doi:10.1371/journal.pone.0114024)

**A**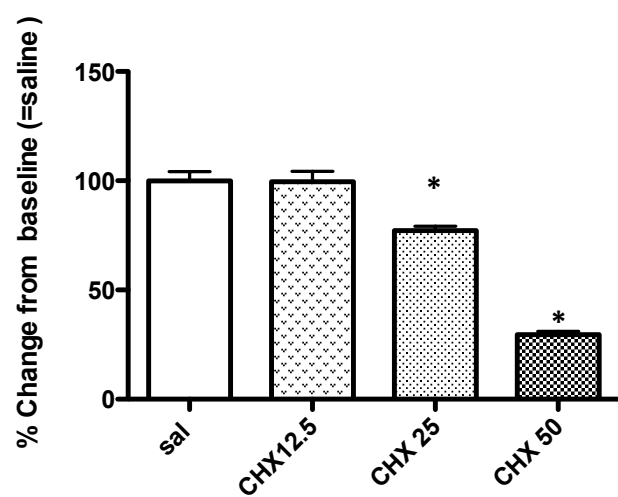**B**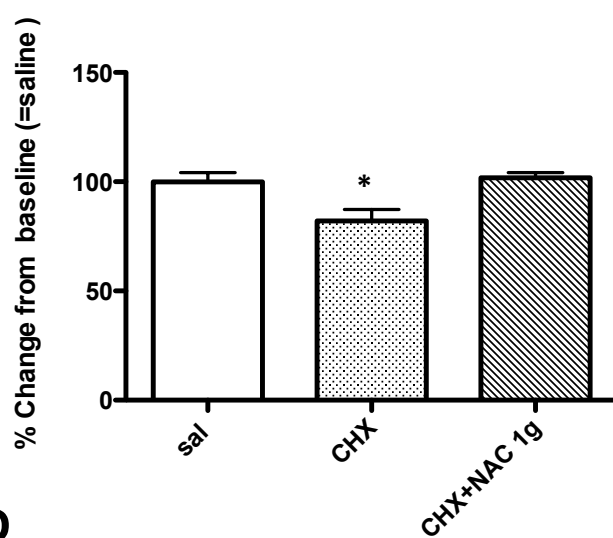**C**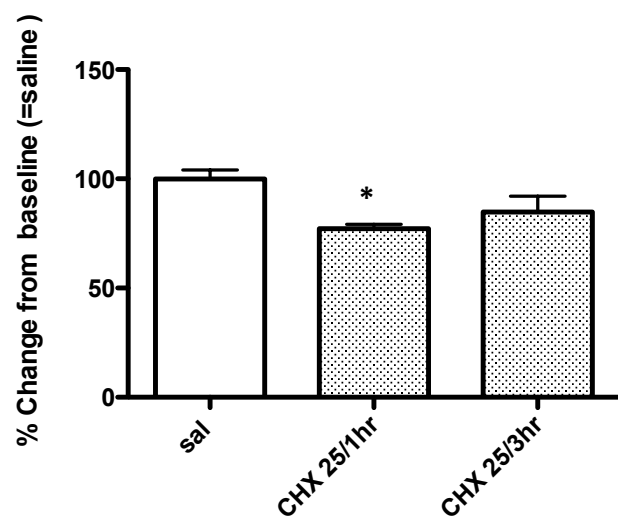**D**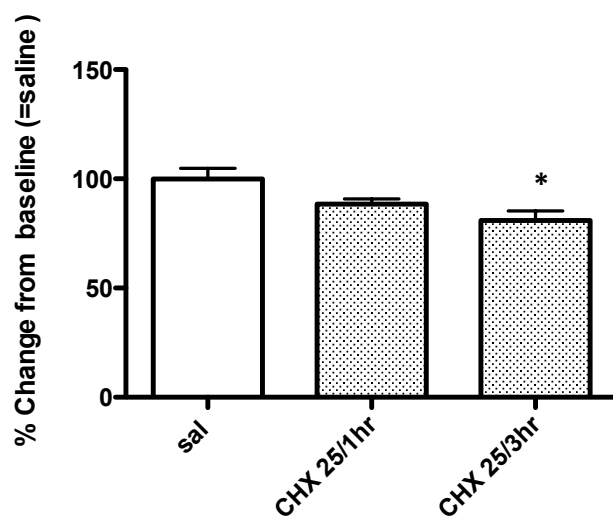**E**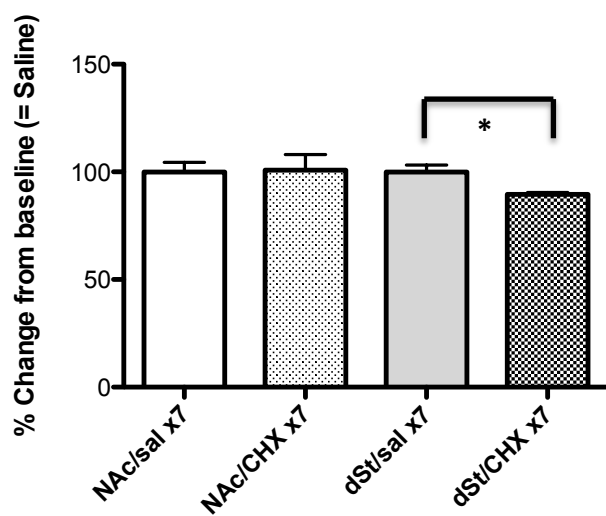**F**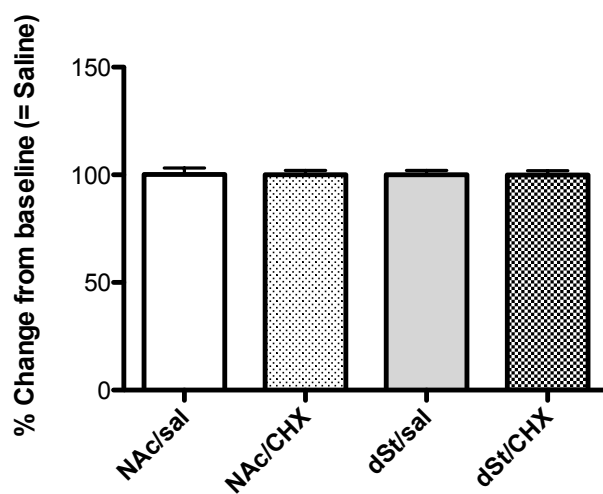

Supplement: S1 Fig — (A) Effects of various doses of CHX on total glutathione levels in the NAc at 1 h after acute CHX administration. Data are represented as relative ratios of the concentration of total glutathione after an acute administration of varying doses of CHX (12.5, 25, and 50 mg/kg, i.p.) to that of saline (N = 4–5 each: *p < 0.05 vs. saline). (B) Effects of an excess amount of the glutathione precursor N-acetylcysteine on total glutathione levels in the NAc when co-administrated with CHX. N-acetylcysteine (1 g/kg) was i.p. administrated 1.5 h before acute injection of 25 mg/kg CHX (N = 4–5: *p < 0.05, vs. saline). (C-D) Temporal effects of an acute CHX administration (25 mg/kg) on the total glutathione levels in the NAc (C; N = 4–5) and dSt (D; N = 5). Data are represented as the relative ratios of the concentrations of total glutathione at 1 and 3 h after the acute administration of 25 mg/kg of CHX to that at 1 h after acute administration of saline (*p < 0.05, vs. saline). (E–F) Effects of repeated CHX administration for 7 days on total glutathione levels in the NAc and dSt at 1 day (E; N = 5 each; dSt, *p < 0.05, vs. saline) or 3 weeks (F; N = 8 each) after the final CHX administration. Data are represented as relative ratios compared with the concentration of total glutathione after repeated saline administration for 7 days in the same region of the brain. Error bars represent SEM. (PDF) [file pone.0114024.s001.pdf]

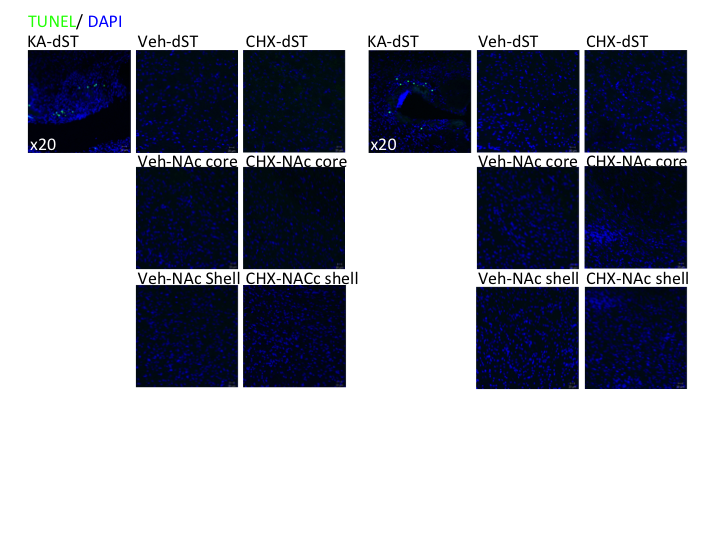

Supplement: S2 Fig — Animals were perfused on the day following the last Vehicle/CHX administration. The brain sections from the core and shell subregions of the NAc and the dSt were compared with sections of the dSt where kainic acid was microinjected. KA: kainic acid, Veh: vehicle. Green: TUNEL, Blue: DAPI. (TIFF) [file pone.0114024.s002.tiff]

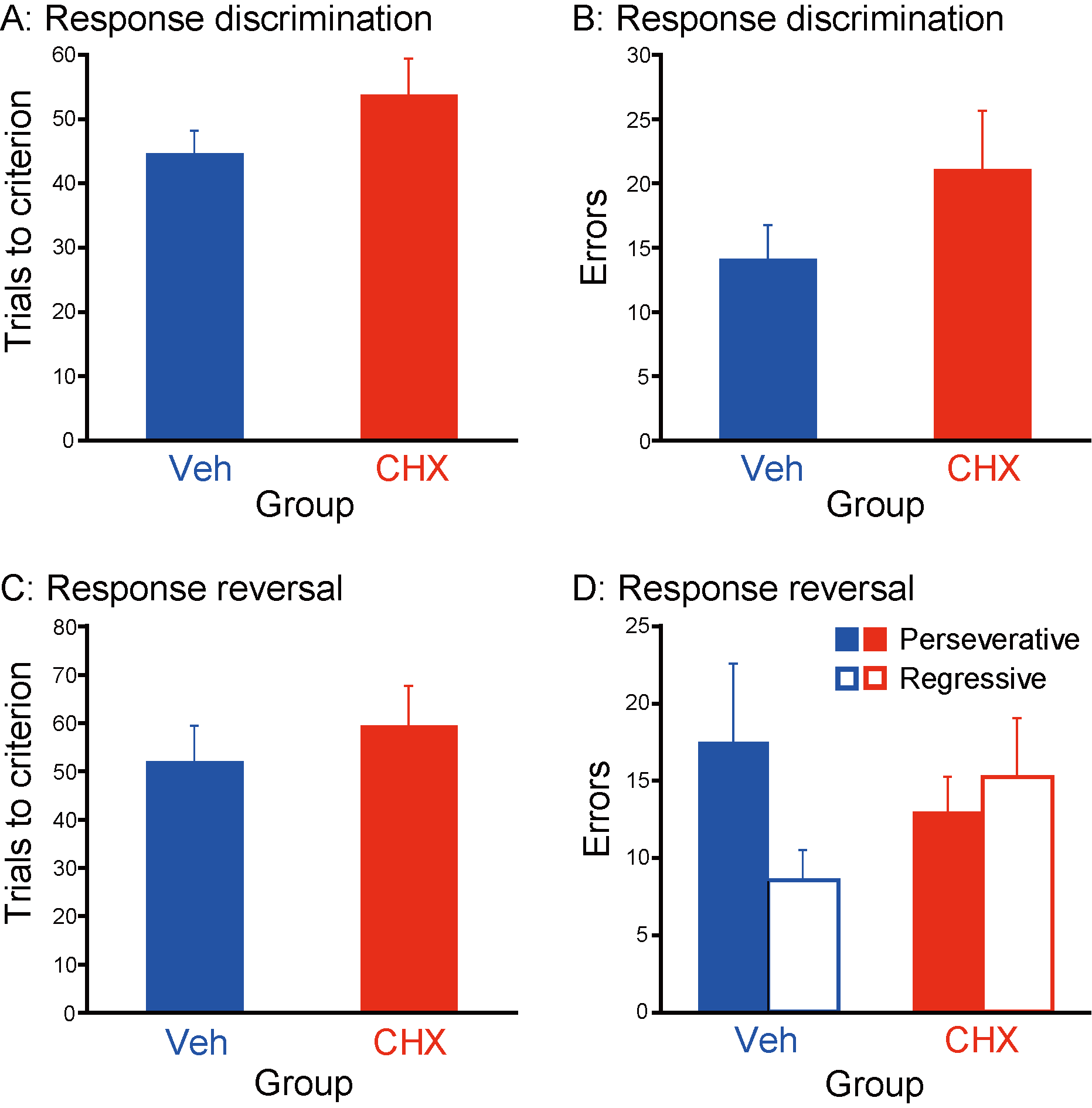

Supplement: S3 Fig — (A) Mean number of trials to reach the criterion of the response discrimination training. A one-way ANOVA (Group: vehicle vs. CHX) did not reveal any significant group difference (F[1, 16] = 1.67). (B) Mean number of errors committed during the response discrimination training without a significant group difference (F[1, 16] = 1.58). (C) Mean number of trials to reach the criterion of the response reversal learning without a significant group difference (F[1, 16] < 1). (D) Mean numbers of the two error subtypes, perseverative and regressive, recorded during the response reversal learning. A two-way ANOVA, 2 (Group: vehicle vs. CHX) × 2 (Error type: perseverative vs. regressive), did not demonstrate significant main effects of Group and Error type (Fs[1, 16] < 1) or interaction between factors (F[1, 16] = 2.10). Data represent the mean + SEM. (TIF) [file pone.0114024.s003.tif]

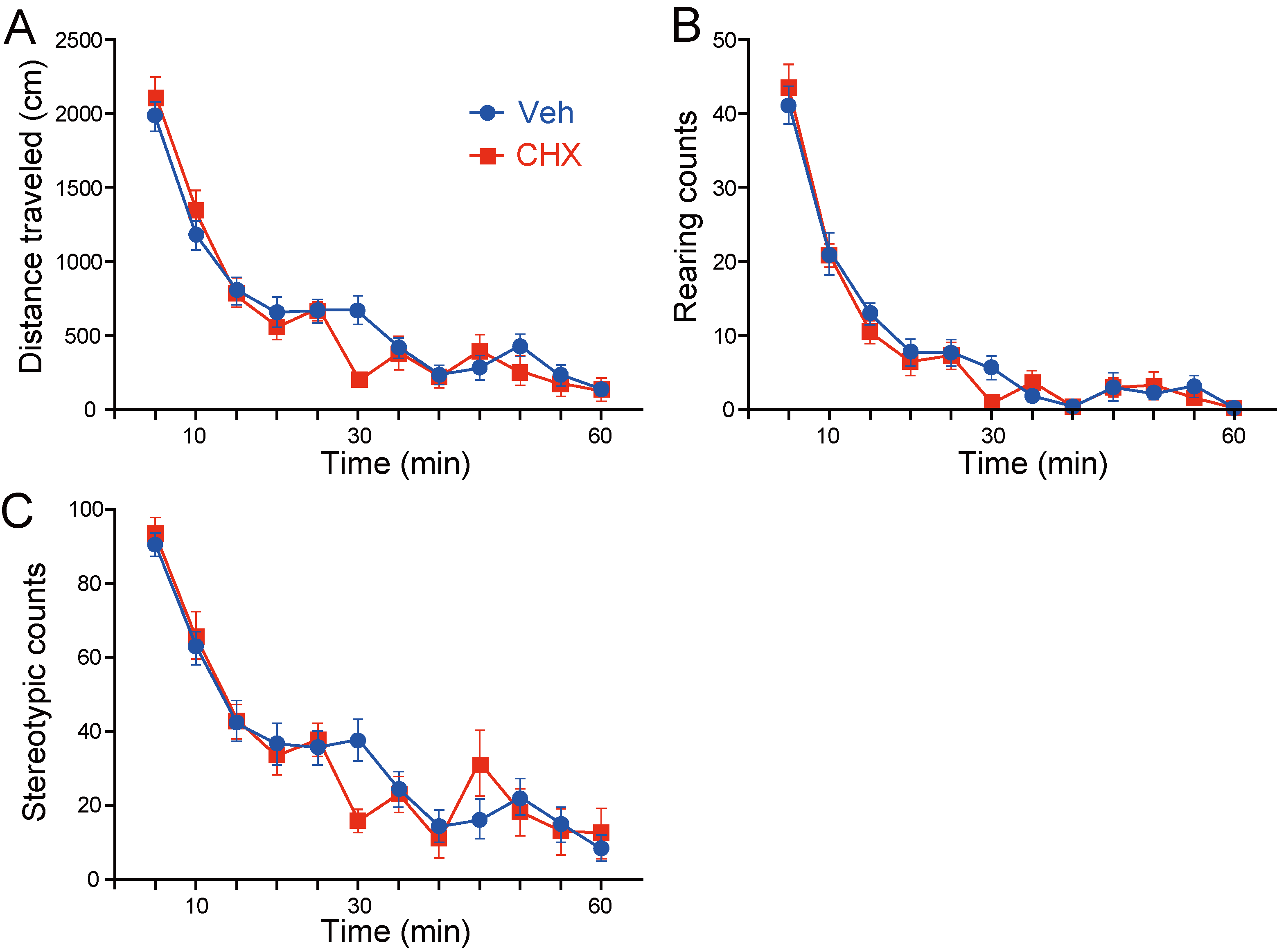

Supplement: S4 Fig — The spontaneous activities in actimeter, locomotor activity (A), rearing counts (B), and stereotypic counts (C) were monitored on the preceding day of the acute cocaine administration (apparatus habituation training, 25 days after repeated vehicle/CHX administration). Separate two-way ANOVAs, 2 (Group) × 12 (Time bin), revealed only significant main effects of Time bin (F[11, 176] = 79.77, 121.16, and 43.87; ps < 0.001) for locomotor activity, rearing, and stereotypy, respectively, without the Group effects (main effect or interactions: Fs < 1.72). Data represent the mean ± SEM. (TIF) [file pone.0114024.s004.tif]

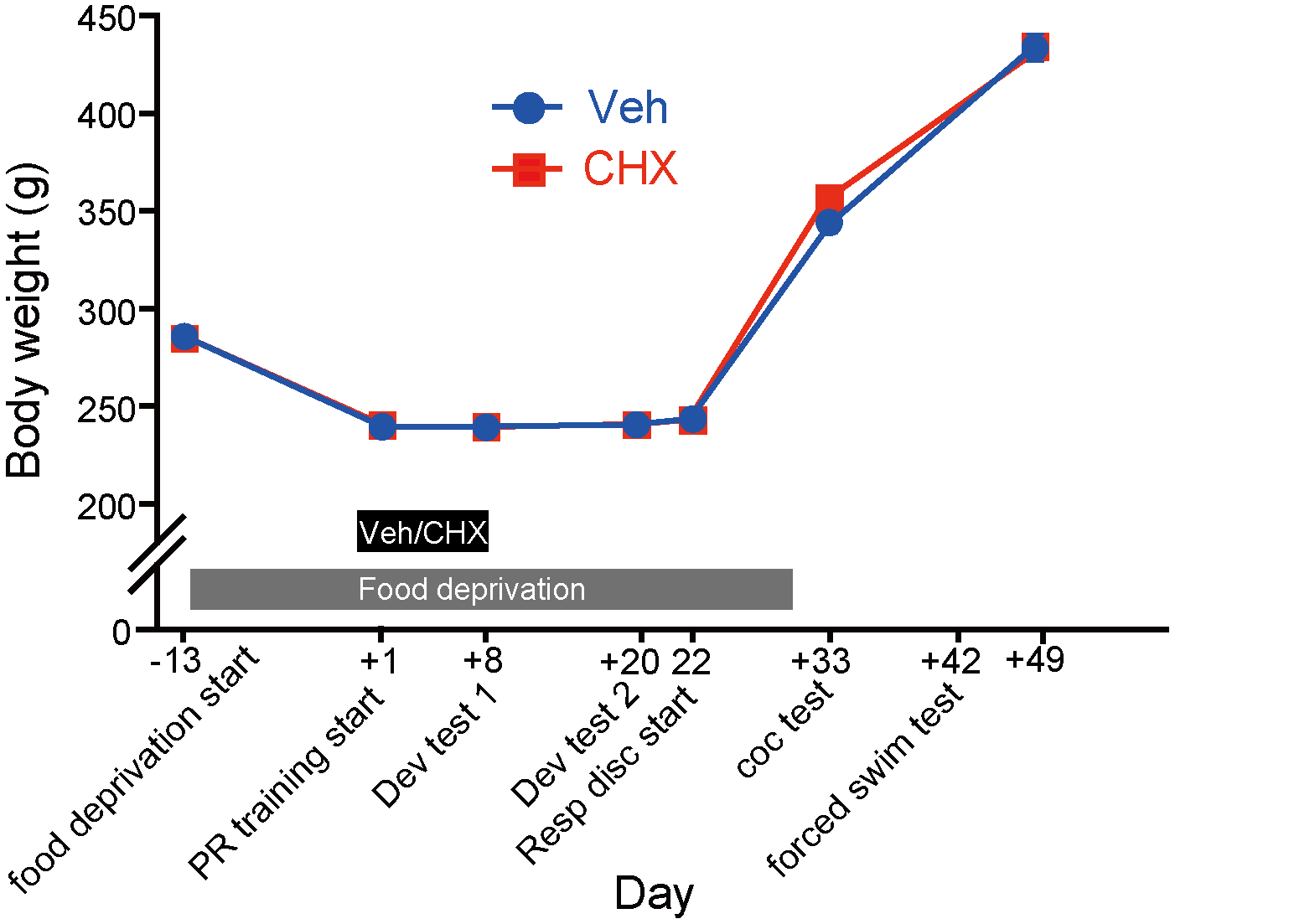

Supplement: S5 Fig — Robust group differences were not detected during repeated CHX administration (7 days), food deprivation, or subsequent ad-libitum feeding. Analysis using two-way ANOVA of 2 (Group) × 7 (Time point) revealed only a significant main effect of Time point, F(6, 96) = 1560.48, p < 0.001. Data represent the mean ± SEM. (TIF) [file pone.0114024.s005.tif]

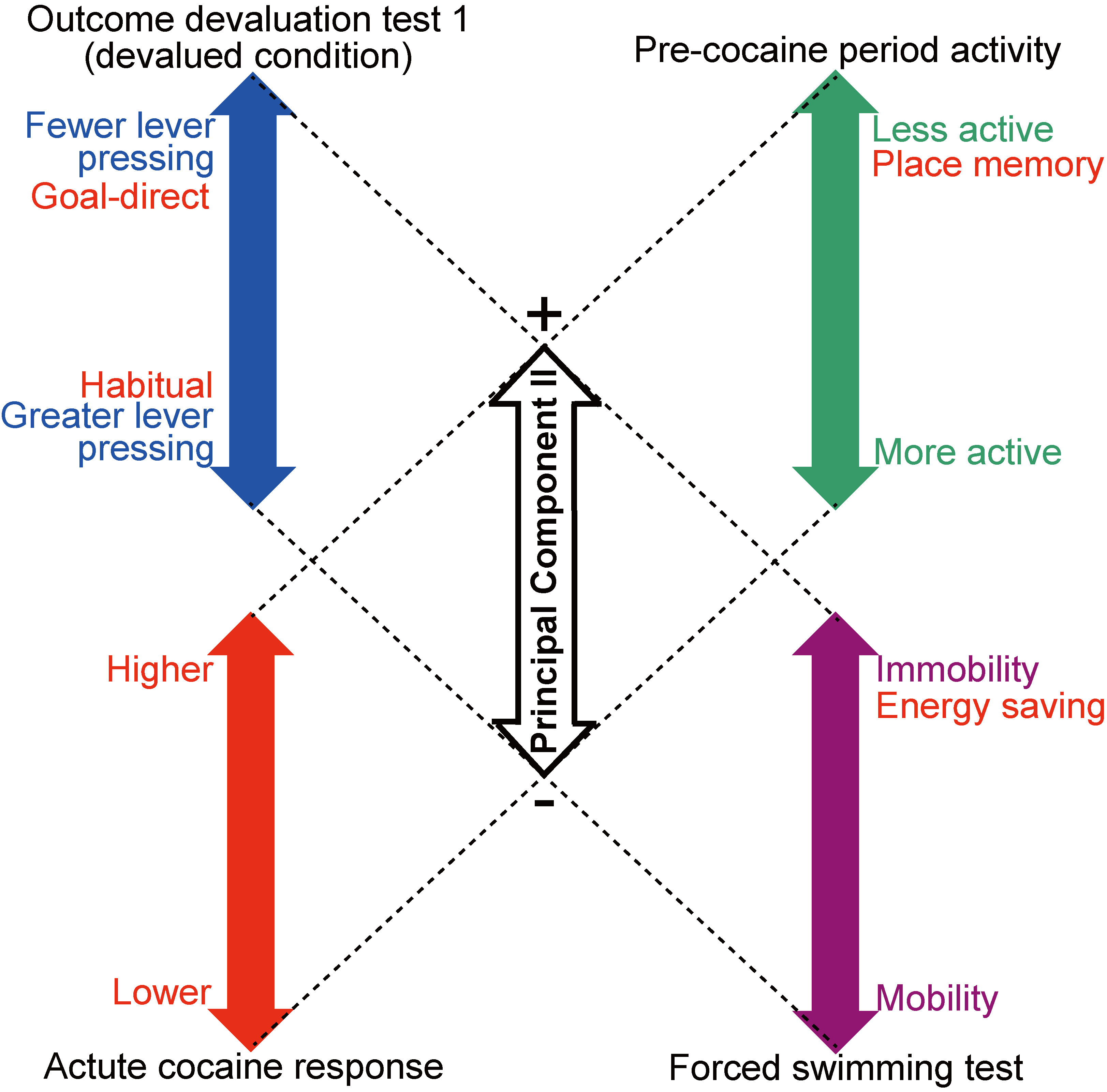

Supplement: S6 Fig — PC II accounted for 20.5% of the variance. This component was negatively loaded by the response rate in the devalued condition of devaluation test 1 as well as open field activity before acute cocaine injection, and positively loaded by open field activity after acute cocaine injection as well as the immobility ratio of the forced swimming test. Analysis of the PC scores calculated for individual animals in Groups Veh and CHX revealed that the scores of animals in Group CHX were significantly lower on PC II than those of animals in Group Veh, t(16) = 2.96, p < 0.05. (TIF) [file pone.0114024.s006.tif]
