# Supplementary material for: Repeated Exposure of Adult Rats to Transient Oxidative Stress Induces Various Long-Lasting Alterations in Cognitive and Behavioral Functions
Source: PLoS One. 2014 Dec 9;9(12):e114024. doi: 10.1371/journal.pone.0114024 (PMC4260961; doi:10.1371/journal.pone.0114024)
Supplement: S1 Table — (DOCX) [file pone.0114024.s007.docx]

**Table S1.** **Intercorrelations (Pearson correlation coefficient) between behavioral phenotypes.**

|  | 1 | 2 | 3 | 4 | 5 | 6 | 7 | 8 | 9 | 10 | 11 | 12 | 13 | 14 | 15 | 16 | 17 | 18 |
| --- | --- | --- | --- | --- | --- | --- | --- | --- | --- | --- | --- | --- | --- | --- | --- | --- | --- | --- |
| 1. Prg 1 |  |  |  |  |  |  |  |  |  |  |  |  |  |  |  |  |  |  |
| 2. Prg 7 | .45 |  |  |  |  |  |  |  |  |  |  |  |  |  |  |  |  |  |
| 3. Dvl 1 (nondev) | -.14 | .00 |  |  |  |  |  |  |  |  |  |  |  |  |  |  |  |  |
| 4. Dvl 1 (dev) | .03 | -.36 | .15 |  |  |  |  |  |  |  |  |  |  |  |  |  |  |  |
| 5. Dvl 2 (nondev) | -.15 | -.19 | .47 | .00 |  |  |  |  |  |  |  |  |  |  |  |  |  |  |
| 6. Dvl 2 (dev) | -.30 | -.46 | .20 | .41 | .36 |  |  |  |  |  |  |  |  |  |  |  |  |  |
| 7. Dsc er | .28 | .53 | -.24 | .01 | -.58 | -.35 |  |  |  |  |  |  |  |  |  |  |  |  |
| 8. Rv psv er | .25 | .44 | .08 | .01 | .20 | .14 | .05 |  |  |  |  |  |  |  |  |  |  |  |
| 9. Rv rgr er | .01 | .04 | .16 | -.10 | .18 | -.21 | .13 | -.15 |  |  |  |  |  |  |  |  |  |  |
| 10. Nvl dst | -.28 | -.35 | -.04 | .15 | .30 | .44 | -.43 | .06 | .04 |  |  |  |  |  |  |  |  |  |
| 11. Nvl r | -.02 | -.19 | -.01 | .32 | .21 | .25 | -.33 | .05 | .22 | **.83***^**^* |  |  |  |  |  |  |  |  |
| 12. Nvl str | -.16 | -.09 | -.16 | .13 | .08 | .29 | -.31 | .18 | .12 | **.87***^**^* | **.78***^*^* |  |  |  |  |  |  |  |
| 13. Pre-coc dst | .12 | -.08 | -.14 | .53 | .14 | .30 | .01 | .37 | .23 | .51 | .58 | .58 |  |  |  |  |  |  |
| 14. Pre-coc r | .37 | -.02 | -.11 | .22 | .19 | .21 | .03 | .29 | .63 | .26 | .48 | .36 | .68 |  |  |  |  |  |
| 15. Pre-coc str | .21 | .09 | -.12 | .33 | .20 | .19 | .11 | .40 | .23 | .51 | .52 | .54 | **.94***^**^* | .61 |  |  |  |  |
| 16. Post-coc dst | -.25 | -.30 | -.05 | -.28 | .44 | .50 | -.58 | .18 | -.13 | .62 | .33 | .44 | -.03 | .08 | .01 |  |  |  |
| 17. Post-coc r | -.04 | -.25 | -.17 | -.30 | .46 | .27 | -.50 | .06 | .32 | .53 | .54 | .41 | .16 | .51 | .15 | .73 |  |  |
| 18. Post-coc str | -.24 | -.41 | -.17 | -.26 | .19 | .44 | -.48 | -.06 | -.05 | .55 | .31 | .44 | -.08 | .14 | -.11 | **.89***^**^* | .68 |  |
| 19. Im | -.26 | .04 | -.34 | -.40 | .06 | .04 | -.13 | .39 | -.40 | .27 | .04 | .13 | -.07 | -.24 | .05 | .48 | .22 | .28 |

*Notes:* Prg = progressive ratio instrumental training; Dvl = response rate during outcome devaluation test; nondev = non-devalued condition; dev = devaluation condition; Dsc er = number of errors in response discrimination training; Rv psv er = number of perseverative errors in response reversal training; Rv rgr er = number of regressive errors in response reversal training; Nvl = activity monitoring in a novel environment; Pre-coc = period prior to acute cocaine administration; Post-coc = period after acute cocaine administration; dst = distance traveled; r = number of rearing; str = number of stereotypy; Im = immobility ratio in forced swimming. ^*^*p*<.05 (Bonferroni’s corrected *α*-value of .00029); ^**^*p*<.01 (corrected *α*-value of .000059).
