# Supplementary material for: Repeated Exposure of Adult Rats to Transient Oxidative Stress Induces Various Long-Lasting Alterations in Cognitive and Behavioral Functions
Source: PLoS One. 2014 Dec 9;9(12):e114024. doi: 10.1371/journal.pone.0114024 (PMC4260961; doi:10.1371/journal.pone.0114024)
Supplement: S2 Table — (DOCX) [file pone.0114024.s008.docx]

**Table S2. Principal component scores calculated for each animal in Groups Veh and CHX.**

|  | | Principal components | | | | |
| --- | --- | --- | --- | --- | --- | --- |
| Animal | | **I** | **II** | **III** | **IV** | **V** |
| *Group Veh* |  | | | | | |
| #1 | | 1.00 | 1.00 | -1.10 | .68 | -.39 |
| #2 | | 2.47 | 2.18 | -2.31 | 1.80 | 2.99 |
| #3 | | .32 | -.64 | 2.30 | -.53 | 1.89 |
| #4 | | -1.52 | 2.63 | 1.27 | -.07 | -.76 |
| #5 | | -.44 | 2.84 | -.89 | -1.66 | -1.35 |
| #6 | | 3.29 | -.53 | -2.39 | -1.66 | -.48 |
| #7 | | -3.95 | -1.27 | -1.57 | 2.36 | .08 |
| #8 | | -2.35 | 2.42 | .88 | .14 | -.26 |
| #9 | | 2.06 | .74 | .52 | -.15 | -.12 |
| Mean | | .10 | 1.04 | -.37 | .10 | .18 |
| SEM | | .75 | .49 | .52 | .43 | .43 |
| *Group CHX* | |  | | | | |
| #1 | | -.42 | -3.31 | -.66 | -1.61 | 1.11 |
| #2 | | -.98 | -.80 | 1.58 | -.42 | .49 |
| #3 | | -.26 | -3.03 | -.26 | 1.87 | -1.23 |
| #4 | | -1.48 | -.31 | -1.51 | -.70 | -1.89 |
| #5 | | 3.35 | -.53 | .96 | -.66 | -.59 |
| #6 | | -1.23 | -1.45 | -.33 | -.58 | -.18 |
| #7 | | -1.34 | -.78 | .86 | -1.49 | 1.32 |
| #8 | | -2.10 | 1.31 | .47 | .37 | .76 |
| #9 | | 3.56 | -.49 | 2.18 | 2.32 | -1.38 |
| Mean | | -.10 | -1.04 | .37 | -.10 | -.18 |
| SEM | | .66 | .44 | .37 | .43 | .37 |
