# Supplementary material for: Repeated Exposure of Adult Rats to Transient Oxidative Stress Induces Various Long-Lasting Alterations in Cognitive and Behavioral Functions
Source: PLoS One. 2014 Dec 9;9(12):e114024. doi: 10.1371/journal.pone.0114024 (PMC4260961; doi:10.1371/journal.pone.0114024)
Supplement: S1 Text — (DOCX) [file pone.0114024.s009.docx]

**Text S1. Supporting materials and methods.**

*Determination of total glutathione concentrations in rat brains.* Animals were decapitated, and the NAc and dSt were immediately dissected and homogenized in 200 µl of ice-cold phosphate-buffered saline (PBS). In total, 40 µl of a tissue homogenate was mixed with an equal volume of PBS containing 2× sodium dodecyl sulfate to determine protein concentration using the BCA Protein Assay Kit (Thermo Fisher Scientific, Waltham, MA, USA), and 40 µl of 25% 5-sulfosalicilic acid was added to the remaining 160 µl, from which the proteins were precipitated with acid. The total amount of glutathione was determined using the GSSG/GSH Quantification Kit according to manufacturer’s protocol (Dojindo Molecular Technologies, Tokyo, Japan).

*Stereotaxic surgery, microinjection of kainic acid, and in situ detection of apoptosis.* The dSt was microinjected with kainic acid to serve as a positive control for histochemical analysis of apoptosis using the TdT-mediated dUTP nick end-labeling (TUNEL) assay, as described previously [1]. Rats (350–390 g) were anesthetized with ketamine hydrochloride (87.5 mg/kg, i.p.) and xylazine hydrochloride (5 mg/kg, i.p.) and then placed in a stereotaxic instrument (Narishige, Tokyo, Japan). Guide cannulas (26 Ga stainless steel tubing, 10.0 mm in length) were bilaterally implanted and aimed at the dorsolateral striatum (anteroposterior: −0.12 to +1.2 mm; mediolateral: −3.8 to +3.5 mm; dorsoventral: −4.2 to −4.0 mm), which were secured with dental cement. After 3 days of recovery, the rats were microinjected with kainic acid (Tocris, Bristol, UK; 2.5 nmol/µl) bilaterally via injector needles (33 Ga) that protruded 0.6 mm past the end of the guide cannulas, at a rate of 0.5 µl/min for 2 min using a microsyringe pump. The injectors were left in place for an additional 90-s period to enable diffusion from the injection sites. Seventy-two hours after the microinjection, the rats received an overdose of sodium pentobarbital (200 mg/kg, i.p.) and were transcardially perfused with PBS for 10 min and then 4% paraformaldehyde for 20 min. The brains were removed and postfixed overnight before transfer immersion in 30% (wt/vol) sucrose in PBS. Coronal sections (20 µm) were cut using a cryomicrotome. The other rats were perfused the day after the last treatment comprising 7 consecutive daily administrations of saline or CHX. TUNEL histochemistry was performed using *In Situ* Apoptosis Detection Kit (Takara, Tokyo, Japan) according to the manufacturer’s protocol.

*Procedures for response discrimination and reversal learning.* Training and testing were conducted as described previously [2-3] with minor modifications. In brief, on the day following the last outcome devaluation test, rats (N = 9 for both Group Veh and CHX) were retrained under an FR 1 schedule until reaching to a criterion of 50 lever presses in 30 min, first for a lever on one side and then another one on the other side. For each rat, the outcome that had been previously employed as the lever press-contingent outcome during the PR training was employed as the reward. On the following days, rats were familiarized with the insertion and retraction of levers and were trained to press them within 10 s of insertion. A session comprised totally 90 trials, and each of which began every 20 s by turning on the house light and inserting one of the two levers into the chamber. If the rat failed to press the lever within the 10-s period, the lever was retracted, the chamber was darkened, and the trial was scored as an omission. If the rat responded within 10 s, the lever was retracted, a single reward was immediately delivered, and the light remained on for another 4 s. The left or right lever was presented in a random order with counterbalancing in number. Each animal was required to achieve a criterion of less than 5 omissions over the 90 trials to proceed to the next stage. The response discrimination training commenced on the following day. In the response discrimination training, the animals were required to respond consistently to one of the two levers (left/right, counterbalanced across animals). A session began with both levers retracted in the dark (intertrial interval). A trial began every 20 s by turning on the light and inserting both levers. A response on the correct lever resulted in the retraction of both levers and delivery of the reward; the light remained on for another 4 s, after which the chamber returned to the intertrial state. An incorrect response resulted in the retraction of both levers and turning off the light immediately without reinforcement. Failure to respond on either lever within 10 s was recorded as an omission trial, returning to the intertrial state. For each animal, trials continued until it received a minimum of 30 trials followed by an achievement of 8 consecutive correct responses. The day after meeting this criterion, the animals underwent a reversal training for the position of the correct lever reversed. This training continued until the rat achieved eight consecutive correct choices. Errors committed during the reversal learning were classified into two subtypes, perseverative and regressive as described previously [2-3]. In brief, the perseverative errors were scored when a rat made an incorrect response by pressing the lever reinforced during the initial response discrimination training. Once a rat made fewer than 10 preservative errors within a block of 16 trials for the first time, all subsequent errors were scored as regressive.

Supporting Information References

1. Nakai M, Qin ZH, Chen JF, Wang Y, Chase TN (2000) Kainic acid-induced apoptosis in rat striatum is associated with nuclear factor-kappaB activation. J Neurochem 74: 647-658.

2. Floresco SB, Block AE, Tse MT (2008) Inactivation of the medial prefrontal cortex of the rat impairs strategy set-shifting, but not reversal learning, using a novel, automated procedure. Behav Brain Res 190: 85-96.

3. Haluk DM, Floresco SB (2009) Ventral striatal dopamine modulation of different forms of behavioral flexibility. Neuropsychopharmacology 34: 2041-2052.
