# Supplementary material for: Correction: Repeated Exposure of Adult Rats to Transient Oxidative Stress Induces Various Long-Lasting Alterations in Cognitive and Behavioral Functions
Source: PLoS One. 2015 Jun 29;10(6):e0132171. doi: 10.1371/journal.pone.0132171 (PMC4488308; doi:10.1371/journal.pone.0132171)
Supplement: S2 File — (PDF) [file pone.0132171.s002.pdf]

RESEARCH ARTICLE

# Repeated Exposure of Adult Rats to Transient Oxidative Stress Induces Various Long-Lasting Alterations in Cognitive and Behavioral Functions

**Yoshio Iguchi*, Sakurako Kosugi, Hiromi Nishikawa, Ziqiao Lin, Yoshio Minabe, Shigenobu Toda**

Department of Psychiatry and Neurobiology, Kanazawa University School of Medicine, Kanazawa, Ishikawa, Japan, 980-8641

* yiguchi@med.kanazawa-u.ac.jp

OPEN ACCESS

**Citation:** Iguchi Y, Kosugi S, Nishikawa H, Lin Z, Minabe Y, Toda S (2014) Repeated Exposure of Adult Rats to Transient Oxidative Stress Induces Various Long-Lasting Alterations in Cognitive and Behavioral Functions. PLoS ONE 9(12): e114024. doi:10.1371/journal.pone.0114024

**Academic Editor:** Thomas H. J. Burne, University of Queensland, AUSTRALIA

**Received:** July 11, 2014

**Accepted:** November 4, 2014

**Published:** December 9, 2014

**Copyright:** © 2014 Iguchi et al. This is an open access article distributed under the terms of the Creative Commons Attribution License, which permits unrestricted use, distribution, and reproduction in any medium, provided the original author and source are credited.

**Data Availability Statement:** All relevant data are within the paper and its Supporting Information files.

**Funding:** This study was supported by Grants-in-Aids from the Ministry of Education, Culture, Sports and Technology, Japan (20452097 and 25860994 to YI; 24791208 to SK; 26860920 to HN; 25461727, 25116510, and 26120712 to ST), Eli Lilly (to YI), Japan Foundation for Neuroscience and Mental Health (to YI), Takeda Research Foundation (to ST) and Matsubara Saburoh Memorial Research Foundation for Psychiatry (to SK, ZL and YI). The funders had no role in study design, data collection

## Abstract

Exposure of neonates to oxidative stress may increase the risk of psychiatric disorders such as schizophrenia in adulthood. However, the effects of moderate oxidative stress on the adult brain are not completely understood. To address this issue, we systemically administrated 2-cyclohexen-1-one (CHX) to adult rats to transiently reduce glutathione levels. Repeated administration of CHX did not affect the acquisition or motivation of an appetitive instrumental behavior (lever pressing) rewarded by a food outcome under a progressive ratio schedule. In addition, response discrimination and reversal learning were not affected. However, acute CHX administration blunted the sensitivity of the instrumental performance to outcome devaluation, and this effect was prolonged in rats with a history of repeated CHX exposure, representing pro-depression-like phenotypes. On the other hand, repeated CHX administration reduced immobility in forced swimming tests and blunted acute cocaine-induced behaviors, implicating antidepressant-like effects. Multivariate analyses segregated a characteristic group of behavioral variables influenced by repeated CHX administration. Taken together, these findings suggest that repeated administration of CHX to adult rats did not cause a specific mental disorder, but it induced long-term alterations in behavioral and cognitive functions, possibly related to specific neural correlates.

## Introduction

Stressors evoke various adaptive or maladaptive responses in individuals that may strengthen or weaken their response, respectively, to forthcoming stress in the near or distant future [1–2]. Many psychiatric disorders of humans, such as schizophrenia, major depression, posttraumatic stress disorder, and drug addiction, can be triggered, reversed, or exacerbated when there is unexpected excessive exposure to diverse stressors or when individuals are vulnerable to these stressors [3–5].

and analysis, decision to publish, or preparation of the manuscript.

**Competing Interests:** The authors have received funding from a commercial source, Eli Lilly. However, the authors declare that this does not alter their adherence to all PLOS ONE policies on sharing data and materials.

A traditional approach to study stress-induced psychiatric disorders was to investigate the relationship between each stressor and each evoked phenotype or pathophysiological response (e.g., social defeat stress and depression). However, one major pitfall of this strategy is the difficulty of dealing with the diversity of stress and the redundancy of its outcome. Humans or animals are often simultaneously exposed to various stressors that generate overlapping responses that are difficult to distinguish from each other. For example, fatigue is generally considered a hallmark of physical stress, but it can be induced by psychological stresses, including social defeat. Depressive mood can be induced by both social defeat and lack of sleep. In addition, a stressor induces various signaling responses (including the hypothalamic—pituitary—adrenal axis, inflammatory reactions, oxidative stress, and the redox system), which mediate stressors and pathological and/or adaptive phenotypes but evoke intertwined biochemical interactions among them [6]. As a result, the relationship between a cause (stressor) and an outcome (symptom or phenotype) is extremely complicated and far from stoichiometric.

To study the roles of stress in animal models of psychiatric disorders in a simplified manner, we used oxidative stress. Oxidative stress has been proposed as a major intracellular mediator for most primary stressors, including physical stress [7] or social stress [8], and it induces relatively characterized and quantifiable biochemical reactions [9]. The relevance of oxidative stress has been repeatedly demonstrated in both clinical and preclinical studies. For example, prenatal or neonatal exposure to oxidative stress caused by infection or a lack of a redox system is proposed to increase the risk of schizophrenia in adolescents [10–11]. However, in cases of major depression or drug addiction, critical exposure to oxidative stress may occur in adolescence or adulthood. Thus, the pathological significance of oxidative stress in adulthood must be completely addressed by both preclinical and clinical studies.

A promising approach for studying the relevance of oxidative stress to brain functions is to utilize animals lacking genes encoding enzymes required for glutathione synthesis because glutathione is one of the most abundant antioxidants in the body [12–13]. One of the most representative molecules causing oxidative stress, reactive oxygen species (ROS), is continuously generated in the brain, which consumes more oxygen than other organs (approximately 20% in the resting state), and the concentration of glutathione in the organs is tightly regulated to counterbalance ROS generation [14]. Thus, any manipulation to reduce glutathione will result in an immediate imbalance of the equilibrium between ROS production and anti-oxidative systems, leading to an immediate increase in ROS. However, using this genetic tool, it is extremely difficult to mimic intermittent stress loading that animals typically encounter in the natural world, and a constitutive non-physiological decrease in glutathione levels may induce stronger neurotoxicity that may cause a neurodegenerative disease-like, rather than a neuropsychiatric disease-like, condition. It is also difficult to distinguish between the direct and indirect effects of oxidative stress (e.g., the consequence of an oxidative stress-induced redox reaction) because of the inability to observe sequential alterations that occur after exposure to stress.

Therefore, in this study, we used a pharmacological method that directly induces moderate and intermittent oxidative stress accompanied by minimal concomitant psychological and physical stress. Among several pharmacological approaches to reduce glutathione in animals [15], 2-cyclohexen-1-one (CHX) binds and enhances the rapid degradation of glutathione and temporally reduces the level of glutathione. In addition, CHX can be systemically administrated to subjects. Moreover, the pharmacological depletion of glutathione is an established method used to investigate the effect of glutathione on memory performance in the Morris water maze test, suggesting a relevant role of redox balance in cognition [16].

However, the effect of a minimal dose of CHX on the behavior and cognition of adult rodents, particularly when administrated repeatedly, remains almost unknown, except a few studies [17]. In this study, we administrated CHX acutely or repeatedly to adult rats to

investigate the effects of repeated mild, rather than severe, oxidative stress exposure on behavioral or cognitive functions that are primarily mediated by the mesocorticolimbic dopamine (DA) system. The reason why we focused on the system was that this system plays a pivotal role in both the process of decision-making in healthy animals/humans [18] and the pathophysiology of schizophrenia, major depression, and drug addiction [19–20]. In addition, it is vulnerable to oxidative stress [21]. We aimed to identify a specific neural correlate that is sensitive to oxidative stress by employing various behavioral and cognitive tasks and clarify whether this type of exposure to oxidative stress would impair various neural functions in a uniform manner. We report that repeated CHX administration did not necessarily induce a certain type of DA-related mental disorder, but it altered several major variables that appeared to be primarily regulated by the DA system.

## Materials and Methods

### Animals

All protocols for animal care and experimentation followed the Guidelines for Proper Conduct of Animal Experiments (Science Council of Japan, June 2006), and they were approved by the Kanazawa University Institutional Animal Care and Use Committee (Permit Number: AP-132850). Male Sprague—Dawley rats (Japan SLC, Hamamatsu, Japan) 60–65 days of age that weighed 275–300 g at the start of the experiments were used. During behavioral experiments, the rats were housed individually in Plexiglas cages (38 cm × 33.5 cm × 17 cm). For biochemical experiments, the animals were housed in groups of 2–3 in identical cages. These home cages were located in a climate-controlled vivarium with a 12-h/12-h light/dark cycle, and all experimental procedures were conducted during the light cycle (between 8:45 and 20:45 h). All surgeries were performed under ketamine hydrochloride and xylazine hydrochloride anesthesia, and all efforts were made to minimize suffering.

### Apparatus

Instrumental training and testing were conducted in three operant chambers (30 cm × 24 cm × 25 cm; Med Associates, St. Albans, VT, USA) located in a soundproof room. Each chamber was equipped with a dispenser that delivered a 45-mg food pellet (BioServ, Frenchtown, NJ) into a recessed magazine, and a miniature solenoid-activated valve was used to deliver 0.1 ml of a 20% sucrose solution into the magazine. Two retractable levers were located on both sides of the magazine. Illumination of the operant chamber was provided by a single 100 mA incandescent bulb located on the top center of the wall opposite of the levers. Microcomputers equipped with MED-PC software (Med Associates) controlled and recorded behaviors. For outcome devaluation tests, three Plexiglas cages (38 cm × 33.5 cm × 17 cm) with floors covered by wood chips served as the outcome consumption context, and they were placed in the room for instrumental training. Cocaine-induced behaviors were monitored using three photoelectric actimeters (Panlab, Barcelona, Spain). Each actimeter comprised an open field constructed of transparent acrylic plastic (45 cm × 45 cm × 30 cm) under faint illumination (approximately 0.5 lx) with a wood chip-covered floor and two planar square frames in which 16 × 16 infrared beams were arranged to surround the open field. When an animal in the open field interrupted the beams, its location and movements were revealed (5 Hz polling rate). ActiTrack software (Panlab, Holliston, MA, USA) was used to analyze the locomotion, rearing, and stereotyping scores. The forced swimming test employed a glass cylinder (52 cm height × 24 cm diameter) filled with water (24 ± 1°C) to a depth of 26 cm.

## Procedure

**Instrumental learning.** Training on a time-constraint progressive ratio (PR) schedule. Seven days before conducting the behavioral procedures, rats were handled daily and placed on a food-deprivation schedule to maintain them at approximately 85% of their *ad libitum* feeding body weight. They had free access to water in their home cage. The animals (N = 34) were trained with food pellet and sucrose outcomes. One outcome was delivered dependent on lever pressing, whereas another outcome was delivered indifferent to lever pressing (lever press-non-contingent outcome); the latter served as the control condition for the outcome devaluation test. The contingency between the outcome (pellets/sucrose) and the lever used in training (left/right) was counterbalanced among the animals. After two daily 30-min initial training sessions, including apparatus habituation and magazine training, the rats were manually trained with a specific lever with the lever press-contingent outcome (Days −6 to −4). Then, they received three daily training sessions, first on a fixed ratio (FR) of 1, then on an FR-2, and finally on an FR-5 schedule (Days −3 to −1). Each session lasted until the rat received 50 outcomes. After the FR training, the animals underwent daily training sessions on a PR schedule for 7 consecutive days (Days 1–7). The PR schedule was based on exponential progression as follows: 1, 2, 4, 6, 9, 12, 15, 20, 25, 32, 40, 50, etc., calculated using the formula $(5 \times e^{0.2n}) - 5$, rounded to the nearest integer, where $n$ is the position in the sequence of the ratio [22]. Each 60-min daily session began with turning on the light and inserting the lever. The session ended with retracting the lever and turning off the light. The lever press-non-contingent outcome was delivered on a variable time schedule that averaged every 180 s (approximately 20 outcomes were delivered in a 60-min period). A delay prevented this outcome from being delivered within 3 s of each lever press.

Outcome devaluation test. Four outcome devaluation tests were conducted after PR training. Devaluation test 1 commenced 24 h after the last PR training session (Days 8 and 9, N = 34). On each test day, the animals were allowed to consume *ad libitum* either the lever press-contingent (devalued condition) or non-contingent outcome (non-devalued condition) for 1 h in the outcome consumption context as follows: the animals received 25 g of pellets in a metal bowl or 150 ml of sucrose solution in a bottle. Immediately after this feeding period, the animals underwent a 10-min test in extinction with the training lever available. The order of the devalued and non-devalued conditions was counterbalanced across the experimental groups. Around half of the animals (N = 18) were administrated a similar devaluation test 13 days after the last PR training session (test 2, Days 20 and 21). Follow-up devaluation tests were administrated to the remaining animals (N = 16) 6 (test 3, Days 13 and 14) and 11 days (test 4, Days 18 and 19) after the last PR training session. No experimental treatment was administrated to animals between these tests. The schedules for instrumental training, the devaluation tests, as well as other behavioral tests are depicted in Fig 1A.

Response discrimination training and reversal. We assessed the acquisition of response discrimination and its reversal learning of the animals subjected to devaluation test 2 (N = 18). The training commenced on the day following devaluation test 2 and lasted for 5 days (Days 22–26); the procedures are described in S1 Text.

**Cocaine-induced behaviors.** Following the instrumental learning sessions, the food-restriction schedule was discontinued, and animals had free access to food and water in their home cage (Day 26). On Day 32, the day before an acute cocaine administration, each animal (N = 18) was acclimated to the open field of the actimeter for 1 h. On the next day (Day 33), each rat was placed into the open field for 30 min to determine the baseline levels of behaviors without injection. After the 30-min period, each animal received cocaine (15 mg/kg, intraperitoneally [i.p.]), and it was placed in the open field for 120 min. In each session, infrared beams

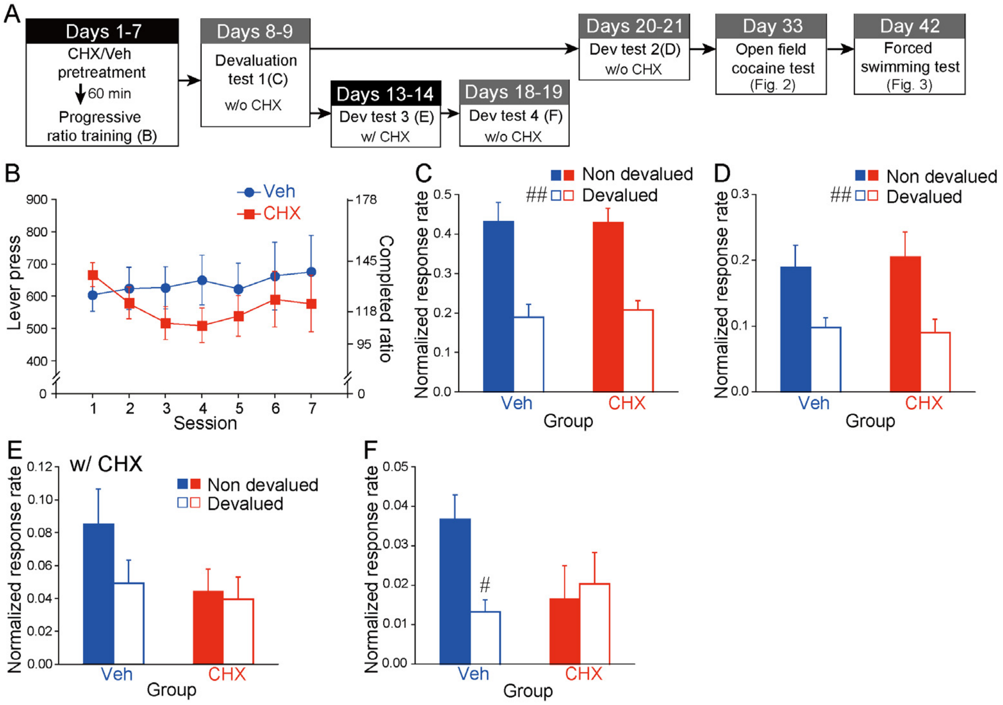


**Fig 1. Effects of repeated 2-cyclohexen-1-one (CHX) administration on motivation and behavioral flexibility in an instrumental learning.** (A) Time course, 7-day time-constraint progressive ratio (PR) instrumental training and outcome devaluation tests. Animals (N = 34) were injected with CHX (25 mg/kg, i.p.) or vehicle 60 min before the start of each training session. (B) The mean number of lever presses (corresponding completed ratio) on PR training. (C–F) Mean response rates (lever presses/min) during the non-devalued versus the devalued condition of the devaluation tests normalized to the response rate of the last day of PR training (C: N = 34, D: N = 18, E & F: N = 16). Data represent the mean ±/+ SEM. $^{\#}p < 0.005$, $^{\#\#}p < 0.001$ compared with the non-devalued condition.

doi:10.1371/journal.pone.0114024.g001

detected horizontal motor activity (distance traveled in cm), rearing (vertical activity over 14 cm), and stereotypy (number of samples where the position of animal was different from its position during the previous sample and equal to its position during the 2nd sample back in time).

**Forced swimming test.** On Day 42, animals (N = 18) were individually placed for 15 min in a glass cylinder containing water. The water was changed after each session, and the cylinder was cleaned to eliminate the possible influence of alarm pheromones left behind by previous animals. The behaviors of the animals were recorded using a video camera (HDR-CX560, Sony, Tokyo, Japan) and analyzed by a trained observer uninformed of the experimental design to rate the behavior as immobility or mobility at the end of each 5-s interval. Immobility was defined as no additional activity other than that required maintaining the rat's head above water with at least three of the rat's paws being immobile [23]. Mobility was defined as climbing, swimming, and diving. The immobility ratio was determined as the ratio of immobility behaviors to the sum of immobility plus mobility behaviors.

## Drugs

CHX (Sigma-Aldrich, St. Louis, MO, USA) was diluted with 0.9% saline and i.p. administrated at 12.5, 25, and 50 mg/kg of body weight to determine glutathione levels in the brain. For behavioral experiments, a dose of 25 mg/kg CHX (for Group CHX) or saline (for Group Veh) was administrated 60 min before each session of the 7 days of PR training. *N*-acetylcysteine (Sigma-Aldrich) was dissolved in saline and i.p. administrated at a dose of 1 g/kg. For outcome devaluation test 3, all animals were injected with CHX at a dose of 25 mg/kg 60 min before the

test (i.e., the *ad-lib* outcome consumption start point). Cocaine hydrochloride (Takeda Pharmaceutical Co., Japan) dissolved in saline was i.p. administrated at a dose of 15 mg/kg.

## Statistical analyses

The numbers of lever presses by animals that received repeated CHX pretreatment (Group CHX) and those that received vehicle (Group Veh) during the 7-day PR training sessions were compared using two-way repeated measures analysis of variance (ANOVA). Because the inter-individual difference in lever presses during PR training (i.e., baseline performance for outcome devaluation tests) was not negligible, we utilized the response rate for the first 10-min period on the last day of the PR training to normalize the data for the 10-min devaluation tests. Two-way repeated measures ANOVA (Group × Value of outcome: non-devalued vs. devalued) was used to evaluate the scores. Animal behaviors in the actimeter (distance traveled and the numbers of rearing and stereotypy behaviors) were recorded at 5-min intervals and analyzed using two-way repeated measures ANOVA. Simple main effect tests were performed as *post hoc* group comparisons when indicated by a significant interaction between factors. One-way ANOVA was used to analyze the immobility ratio of the forced swimming test. The aforementioned statistical analyses were performed using ANOVA 4 on the web (http://www.hju.ac.jp/~kiriki/anova4). The Pearson correlation coefficients ($r$) between the principal behavioral measures and $t$-tests for the correlation coefficients were analyzed using GraphPad Prism ver. 5.04 software (Graph Pad Software Inc., San Diego, CA, USA) with Bonferroni's method for correction of multiple tests. This software was also used for the analyses of biochemical experiments, one- or two-way ANOVAs, and subsequent multiple comparisons by using Student's $t$-test. Principal component analysis (PCA) of the behavioral measures and subsequent $t$-tests (corrected with Bonferroni's method) for the group differences of each principal component (PC) score were performed using R ver. 2.15.3 software [24]. The reliability of all statistical tests was assessed against a type I error rate ($\alpha$) of 0.05.

# Results

## Optimization of the CHX dose for behavioral experiments to minimize drug-induced neurotoxicity

Before determining the effect of acute or repeated CHX administration on various behaviors, we determined the most appropriate dose of CHX (S1 Text). First, we administrated varying doses of CHX i.p. to rats and determined the effect of CHX on the glutathione concentration in the nucleus accumbens (NAc). The concentration of glutathione in the NAc or dorsal striatum (dSt) of the control animals administrated saline was approximately 30–40 nmol/mg, nearly compatible with previous reports that employed similar methods [25–26], and there was no significant difference between these two regions (data not shown). Acute administration of 12.5 mg/kg CHX did not detectably reduce glutathione levels, whereas CHX doses $\geq$ 25 mg/kg induced a significant reduction in total glutathione levels in the NAc, confirming a dose-dependent effect of CHX on total glutathione levels (one-way ANOVA, $F[3, 14] = 73.61$, $p < 0.001$; *post hoc* $t$-test, saline vs. CHX 25 mg, $p < 0.001$; saline vs. CHX 50 mg, $p < 0.0001$; CHX 12.5 mg vs. CHX 25 mg, $p < 0.01$; CHX 12.5 mg vs. CHX 50 mg, $p < 0.0001$; CHX 25 mg vs. CHX 50 mg, $p < 0.0001$; Figure A in S1 Fig). This effect was blocked by an excess amount of the precursor of cysteine, *N*-acetylcysteine (1 g/kg, i.p.) (one-way ANOVA, $F[2, 11] = 6.367$, $p < 0.05$; *post hoc* $t$-test, saline vs. CHX, $p < 0.05$; CHX vs. CHX + NAC, $p < 0.05$; Figure B in S1 Fig). As the supply of cysteine (or *N*-acetylcysteine) is the rate-limiting factor in glutathione synthesis in astrocytes [27], this result further confirmed the expected effect of CHX on total glutathione levels.

We next determined the effect of 25 mg/kg CHX on glutathione turnover. In the NAc, the effect of acute CHX administration was restored after 3 h (one-way ANOVA, $F[2, 11] = 4.62$, $p < 0.05$; *post hoc t*-test, saline vs. CHX 1 h, $p < 0.05$; Figure C in S1 Fig). In contrast, in the dSt, the effect of CHX was detected at 3 h but not 1 h after CHX administration (one-way ANOVA, $F[2, 12] = 5.75$, $p < 0.05$; *post hoc t*-test, saline vs. CHX 3h, $p < 0.05$; Figure D in S1 Fig), suggesting a region-specific difference in glutathione turnover. In consistent with the acute effect of CHX, consecutive CHX administration for 7 days altered the glutathione levels of the dSt, but not of the NAc, versus baseline 24 h after the last CHX session (*t*-test; NAc, $p = 0.9156$; dSt, $p = 0.0143$; Figure E in S1 Fig). However, after a 3-week withdrawal period, there was no significant effect of previous CHX administration on the glutathione levels of the NAc or dSt (*t*-test: NAc, $p = 0.9856$; dSt, $p = 0.9757$; Figure F in S1 Fig).

We then determined whether the administration of 25 mg/kg CHX induced neural cell apoptosis by conducting *in situ* assays of TdT-mediated dUTP-biotin nick end labeling (TUNEL) in the dSt and NAc using kainic acid-induced apoptosis as the positive control (S1 Text). Microinjection of kainic acid into the dSt of naïve rats induced robust TUNEL signals as expected, whereas in the animals that were repeatedly administrated CHX, TUNEL signals were undetectable in the dSt or NAc on the day following the last CHX administration (S2 Fig). Accordingly, we used a CHX dose of 25 mg/kg for behavioral studies because this dose was appropriate for minimizing the adverse effects of CHX, such as prolonged reduction in glutathione levels or apoptosis.

## Effect of repeated CHX administration on instrumental learning

**CHX pretreatment and time-constraint PR instrumental training.** An appetitive instrumental performance trained under a PR schedule has been widely used for investigating the acquisition process of reward-seeking and the motivation required for the learning [28]. By employing this strategy, we first investigated the effect of CHX administrated 60 min before each training session on these two factors, which are essential for effort-based reinforcement learning. Animals readily acquired the PR task from the first training session, and there was no obvious variation in the PR performance without any significant group difference during the seven sessions (Fig 1B). Although we defined the breakpoint as the last ratio measured before 5 min elapsed without any lever press, this breakpoint criterion was not met in most cases. An ANOVA (2 [Group: Vehicle vs. CHX] × 7 [Session]) did not indicate significant main effects (Group: $F[1, 32] < 1$; Session: $F[6, 192] < 1$) or interactions ($F[6, 192] = 1.02$).

**Outcome devaluation tests after 7 days of PR training.** The learning and performance of an instrumental behavior are balanced between two distinct mutually competing processes called goal-directed and habitual processes [29–33]. In addition, the sensitivity of instrumental performance to outcome devaluation is widely used as an index to distinguish goal-directed process from habitual process. In short, as long as an instrumental performance is sensitive to outcome devaluation, it is goal-directed; otherwise, it is habitual [29–33]. To examine whether lever-pressing performance trained with CHX pretreatment is goal-directed or habitual, we evaluated the sensitivity of the animals to outcome devaluation induced by outcome-specific satiation. The devaluation tests performed immediately (test 1; Fig 1C) and 13 days after the last PR training (test 2, Fig 1D) revealed significant sensitivities of the instrumental performance for outcome devaluation in both groups. ANOVAs (2 [Group: Vehicle vs. CHX] × 2 [Value: non-devalued vs. devalued]) revealed a significant main effect of Value for test 1 ($F[1, 32] = 45.78$, $p < 0.001$) and 2 ($F[1, 16] = 49.00$, $p < 0.001$).

**Outcome devaluation tests with CHX challenge.** Next, we determined whether acute administration of CHX would alter the effect of outcome devaluation in both groups. The first

devaluation test (test 3, 6 days after the last PR session) was performed 60 min after a CHX injection (Fig 1E), and the second devaluation test was performed 4 days after test 3 for the same animals to await CHX washout. Test 4 was performed without any CHX administration to determine whether the previous CHX administration induced any long-term effect (Fig 1F). An ANOVA for test 3 (2 [Group: Veh vs. CHX] × 2 [Value]) did not indicate significant main effects (Group: $F[1, 14] = 1.56$; Value: $F[1, 14] = 2.86$) or interaction ($F[1, 14] = 1.67$). However, a similar ANOVA for test 4 revealed a significant interaction between the factors ($F[1, 14] = 6.78$, $p < 0.05$). In addition, a significant simple main effect of Value was revealed in Group Veh ($F[1, 14] = 10.15$, $p < 0.01$). In contrast, the animals in Group CHX did not respond differently to the two Value conditions ($F[1, 14] < 1$).

**Response discrimination training and reversal.** To assess the effect of CHX on flexible behavior in response to changing environmental contingencies, we analyzed the acquisition of response discrimination and learning of its reversal (S1 Text). For the initial discrimination, the animals were trained to consistently press a particular lever between two (left or right) until they reached a criterion of eight consecutive correct choices. On the following day, the animals were trained to press the other lever. Repeated CHX administration did not affect the initial response discrimination or reversal learning (S3 Fig).

## Effect of repeated CHX administration on acute cocaine-induced behaviors

We examined the effects of repeated CHX pretreatment during the 7-day PR training sessions on cocaine-induced behaviors at 26 days after the last CHX administration. Although a single dose of cocaine markedly increased locomotor activity, rearing, and stereotypy for Groups Veh and CHX, repeated CHX administration attenuated these cocaine-induced behaviors (Fig 2A–2C). An ANOVA (2 [Group: Veh vs. CHX] × 24 [Time bin]) for locomotor activity indicated significant main effects of Group ($F[1, 16] = 4.81$, $p < 0.05$) and Time bin ($F[23, 368] = 32.51$, $p < 0.001$), along with a significant interaction between the factors ($F[23, 368] = 3.02$, $p < 0.001$: Fig 2A). ANOVAs for rearing and stereotypy indicated significant main effects of Time bin (for rearing, $F[23, 368] = 13.09$, $p < 0.001$; for stereotypy, $F[23, 368] = 33.30$, $p < 0.001$) and significant interactions between the factors (for rearing, $F[23, 368] = 1.81$, $p < 0.05$; for stereotypy, $F[23, 368] = 1.89$, $p < 0.01$; Fig 2B and 2C). CHX exerted a significant effect on open-field behaviors during the precocaine injection period (Fig 2A). An ANOVA (2 [Group] × 6 [Time bin]) for locomotor activity during the precocaine period revealed a significant main effect of Time bin ($F[5, 80] = 66.95$, $p < 0.001$) and a significant interaction between factors ($F[5, 80] = 2.35$, $p < 0.005$). A subsequent simple main effect test revealed that during the first 5 min, the animals in Group CHX traveled more than those in Group Veh ($F[1, 96] = 7.75$, $p < 0.01$). Similar ANOVAs on rearing and stereotypy during the precocaine period indicated only significant main effects of Time bin (for rearing, $F[5, 80] = 40.44$, $p < 0.001$; for stereotypy, $F[5, 80] = 51.29$, $p < 0.001$; Fig 2B and 2C). The behaviors during 1-h acclimation sessions on one day before acute cocaine administration did not display any significance group differences (ANOVAs, 2 [Group] × 12 [Time bin]; main effect or interactions, $F < 1.72$; S4 Fig), excluding the main effects of Time bin ($Fs[11, 176] = 79.77$, 121.16, and 43.87, $p < 0.001$, for locomotor activity, rearing, and stereotypy, respectively).

## Effect of repeated CHX administration on the forced swimming test

The effect of repeated CHX administration on immobility induced by forced swimming was assessed 35 days after the last CHX administration. Repeated CHX administration significantly reduced immobility (one-way ANOVA [Group: Vehicle vs. CHX], $F[1, 16] = 6.39$, $p < 0.05$; Fig 3).

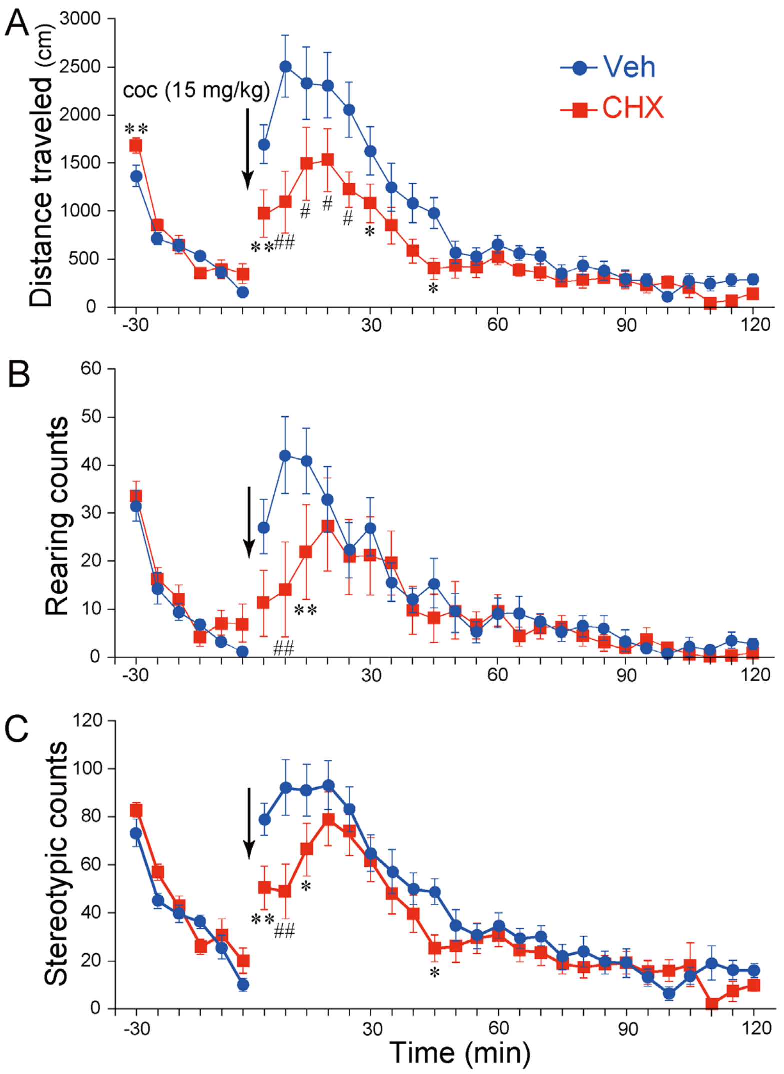


**Fig 2. Effects of repeated 2-cyclohexen-1-one (CHX) administration on behavioral responses to an acute cocaine injection.** (A) Locomotor activity (distance traveled in cm), (B) number of rearings, and (C) number of stereotypic movements before and after an acute injection of cocaine (15 mg/kg intraperitoneally; arrow). The test was conducted 26 days after repeated CHX or vehicle administration (N = 9/each). Data represent the mean ± SEM. **p* < 0.05, ***p* < 0.01, $^{\#}p < 0.005$, $^{\#\#}p < 0.001$ compared with control (Veh).

doi:10.1371/journal.pone.0114024.g002

## Effect of repeated CHX administration on body weight

Repeated CHX administration did affect body weight during the food restriction and subsequent *ad libitum* feeding periods (S5 Fig).

## Multivariate analyses through behavioral variables

To clarify the relationships between the numerous behavioral variables of the two groups and to study the putative neural substrates underlying alterations induced by CHX, we performed principal component analyses (PCAs). S1 Table shows the matrix of Pearson's correlation coefficients between all 19 behavioral variables described previously for the vehicle- and CHX-treated animals (N = 9 each). Based on the correlation matrix, we first conducted a preliminary

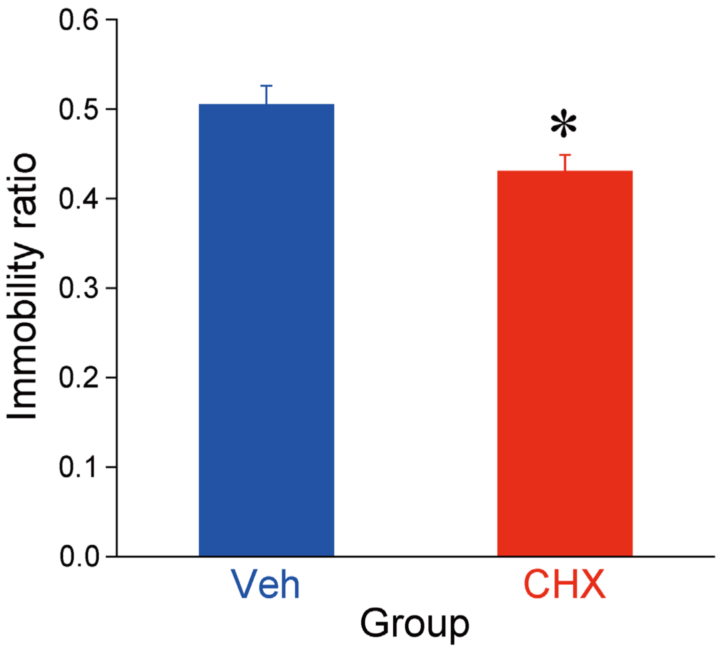


**Fig 3. Effects of 2-cyclohexen-1-one (CHX) on immobility during the forced swimming test.** The test was conducted 35 days after repeated CHX or vehicle administration control (N = 9/each). Data represent the mean + SEM. **p* < 0.05 compared with control (Veh).

doi:10.1371/journal.pone.0114024.g003

PCA to extract the principal behavioral variables and identified 6 principal components (PCs) with eigenvalues > 1 consolidated from the 19 variables, representing 84.6% of the variability (data not shown). We then selected 15 variables with contributions of loading exceeding 0.80 as the principal behavioral variables for secondary PCA, which was conducted with more stringent criteria. The 15 behavioral variables were consolidated into 5 PCs with eigenvalues > 1, representing 84.7% of the variability (Table 1).

PCs I, II, III, IV, and V accounted for 30.0%, 20.5%, 13.1%, 11.2%, and 9.8% of the variance, respectively. For PC I, positively loading behavioral variables with contributions exceeding 0.50 (absolute value) represented spontaneous behaviors in the open field, locomotion, rearing, and stereotypy, suggesting that PC I is likely related to motor functions. For PC II, the behavioral variables loading negatively were the response rate in the devalued condition of devaluation test 1 and the open-field activity prior to acute cocaine injection, whereas the positively loaded variables corresponded to the open-field activity after acute cocaine administration and the immobility ratio in forced swimming test (S6 Fig). PC III was negatively loaded by the number of perseverative errors in reversal learning and the immobility ratio. PC IV was positively loaded by the number of lever presses in the final PR training and the number of regressive errors in reversal learning and negatively loaded by the response rate in the devalued condition of devaluation test 1. PC V was positively loaded by the response rates in the valued condition of devaluation tests 1 and 2.

Finally, we analyzed the PC scores that were calculated for individuals in Groups Veh and CHX on the basis of loadings derived from PCA (S2 Table). Multiple unpaired *t*-tests with Bonferroni's correction revealed that the scores for PCs I, III, IV, and V were similar between the two groups (the largest; $t[16] = 1.08$). In contrast, the scores for the animals in Group CHX were significantly lower for PC II than those for animals in Group Veh ($t[16] = 2.96$, $p < 0.05$; Fig 4).

**Table 1. Results of principal component analysis of 15 principal behavioral variables.**

| | Principal components | | | | | |
|---|---|---|---|---|---|---|
| **Behavioral variable** | **I** | **II** | **III** | **IV** | **V** | **Contribution** |
| **Prg 7** | −0.286 | −0.208 | **−0.575** | **0.558** | −0.113 | 0.780 |
| **Dvl 1 (non-dev)** | −0.134 | −0.128 | 0.434 | 0.354 | **0.698** | 0.836 |
| **Dvl 1 (dev)** | 0.081 | **−0.615** | 0.201 | **−0.551** | 0.347 | 0.849 |
| **Dvl 2 (non-dev)** | 0.439 | 0.081 | 0.309 | 0.433 | **0.573** | 0.810 |
| **Rv psv er** | 0.288 | −0.199 | **−0.655** | 0.388 | 0.354 | 0.827 |
| **Rv rgr er** | 0.235 | −0.378 | 0.485 | **0.509** | −0.418 | 0.867 |
| **Nvl dst** | **0.864** | 0.085 | 0.020 | −0.288 | 0.127 | 0.853 |
| **Nvl str** | **0.794** | −0.096 | −0.130 | −0.234 | −0.076 | 0.717 |
| **Precoc dst** | **0.641** | **−0.703** | −0.172 | −0.167 | 0.026 | 0.963 |
| **Precoc r** | **0.616** | **−0.521** | 0.143 | 0.290 | −0.322 | 0.859 |
| **Precoc str** | **0.624** | **−0.642** | −0.284 | −0.020 | 0.033 | 0.884 |
| **Postcoc dst** | **0.696** | **0.653** | 0.007 | 0.061 | 0.152 | 0.937 |
| **Postcoc r** | **0.756** | 0.355 | 0.209 | 0.297 | −0.226 | 0.881 |
| **Postcoc str** | **0.620** | **0.648** | 0.171 | −0.095 | −0.115 | 0.856 |
| **Im** | 0.255 | **0.524** | **−0.657** | −0.007 | 0.103 | 0.782 |
| **Eigenvalue** | 4.499 | 3.068 | 1.971 | 1.687 | 1.474 | |
| **Contribution** | 0.300 | 0.205 | 0.131 | 0.112 | .098 | |
| **Contribution (cumulative)** | 0.300 | 0.504 | 0.636 | 0.748 | .847 | |

*Notes*: Factor loadings exceeding 0.50 (absolute value) are shown in bold. Prg = progressive ratio instrumental training; Dvl = response rate during the outcome devaluation test; non-dev = non-devalued condition; dev = devalued condition; Rv psv er = number of perseverative errors in reversal training; Rv rgr er = number of regressive errors in reversal training; Nvl = activity monitoring in a novel environment; Precoc = period prior to acute cocaine administration; Postcoc = period after acute cocaine administration; dst = distance traveled; r = number of rearing; str = number of stereotypy; Im = immobility ratio in forced swimming test.

doi:10.1371/journal.pone.0114024.t001

## Discussion

In the present study, we found that acute or repeated CHX administration exerted various effects on several behavioral and cognitive functions of adult rats that were relatively short-lived or prolonged, respectively. Previous studies on acute glutathione depletion by CHX or other molecules such as buthionine sulfoximine or diethylmaleate on a Y-maze or Morris water maze task have suggested a role of glutathione homeostasis in certain learning and memory tasks [16, 34–36]. In this study, we employed a lower concentration of CHX than that employed in those studies (75 mg/kg vs. 25 mg/kg), but this concentration of CHX was sufficient to significantly reduce the levels of total glutathione in the rat NAc and dSt. In this study, we investigated the effect of repeated CHX administration on glutathione levels, but not on other indices of oxidative stress, such as ROS, directly. As mentioned in Introduction, exposure to oxidative stress does not necessarily occur in a sporadic or accidental manner. ROS are endogenously present, but their effect is masked by glutathione, which is also continuously generated [14]. Thus, any alteration in total glutathione content is inevitably expected to produce a concomitant fluctuation of ROS levels, particularly in the brain, although it could be too subtle and transient to monitor. Similar to the relatively mild reduction in glutathione in the present study, it has been reported that during early postnatal period repeated administration of buthionine sulfoximine, which causes a 20% reduction in glutathione levels in the rat brain, results in many cognitive deficits in adulthood [37].

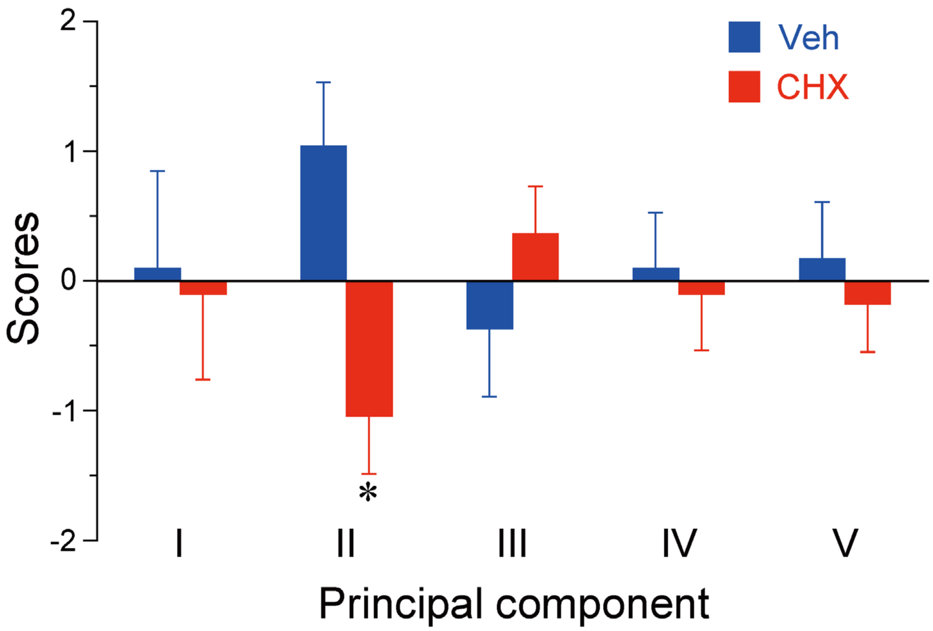


**Fig 4. Principal component scores derived from principal component analysis (PCA) of the behavioral variables.** The scores were calculated for individual animals administrated saline or 2-cyclohexen-1-one (N = 9/each) using loadings derived from PCA. Data represent the mean ± SEM. *$p$ < 0.05 (Bonferroni's corrected $\alpha$-value of 0.01) compared with control (Veh).

doi:10.1371/journal.pone.0114024.g004

We found that repeated CHX administration increased locomotor activity compared with control during the first 5 min of the second apparatus habituation training, which was performed before cocaine was administered acutely (Fig 2A). This finding suggests impaired spatial memory in CHX animals in line with previous reports, as the novelty-induced activity recorded on the previous day (the first habituation session) did not differ between the two groups (S4 Fig).

CHX did not appear to have any significant temporal effect on either the acquisition of instrumental behavior or the motivation to perform it because compared to Group Veh there was no alteration in the instrumental performance in Group CHX during the PR training period (Fig 1B). Moreover, repeated CHX administration did not significantly alter the sensitivity to outcome devaluation immediately or 13 days after the last PR session (Fig 1C and 1D). Once animals acquired the instrumental behavior in the absence of CHX pretreatment (Group Veh), acute CHX administration reduced the sensitivity to outcome devaluation, although this sensitivity was recovered after washout (Fig 1E and 1F). However, in Group CHX, the insensitivity of the instrumental performance to outcome devaluation induced by a CHX challenge was prolonged even after CHX washout (Fig 1E and 1F, Group CHX). Thus, we concluded that the insensitivity of instrumental performance to outcome devaluation was induced by the acute effect of CHX but not by the long-term effect of repeated CHX administration. However, in Group CHX, the restoring process of the original sensitivity to the outcome devaluation from the effect of CHX challenge was delayed, probably due to increased vulnerability to acute oxidative stress, another long-term effect of previous repeated exposures to oxidative stress.

In addition to general acquisition or expression, the acquisition and performance of instrumental behavior are being balanced between two distinct mutually competing intrinsic systems called goal-directed and habitual processes [29–33]. Goal-directed process consists of the formation of an association between an action and the information regarding the outcome

produced by the action (i.e., sensory properties and incentive values of the outcome) and feed-forward control of action performance based on current value of the outcome. On the other hand, habitual process represents reinforcement learning between a behavior and its antecedent environmental stimuli, indifferent to outcome information, leading to stimuli-induced behavior that is insensitive to outcome devaluation. The results of the present study suggest that a single dose of CHX transiently interfered with the expression, but not the acquisition, in goal-directed process. Similar to that finding, previous studies demonstrated that a single episode of stress acutely reduces the sensitivity of human appetitive instrumental performance to outcome devaluation [38–40].

In the present study, despite the fact that stressed animals have demonstrated augmented response to psychostimulants [41], acute cocaine administration attenuated the responses in Group CHX. Although cocaine binds to the DA transporter (DAT) and serotonin and norepinephrine transporters with almost equal affinity [42], its major motor effects are provoked by the blockade of DAT at low or moderate concentrations and a consequent elevation of extracellular DA levels (DA tone) in all terminal fields expressing DAT [43–44]. The extent of DA efflux following psychostimulant administration in the NAc, the terminus of the mesoaccumbal DA pathway, is implicated in hyperlocomotion [44–47]. Meanwhile, increased DA transmission in the dSt, the terminus of the nigrostriatal DA pathway, is associated with augmented species-specific stereotyped behaviors [46, 48–49]. Rearing of rats is often described as a landmark of cocaine-induced stereotyped behaviors [50–51]. Thus, our results suggest that repeated CHX administration interfered with the acute cocaine-induced DAergic signaling pathway in the NAc and dSt.

A plausible neural mechanism underlying the attenuated response to cocaine is that repeated CHX administration decreased the number of functional DATs in the mesoaccumbal/nigrostriatal DAergic pathways. Indeed, DAT knockout (KO) mice do not exhibit an increase in locomotor activity in response to cocaine [43, 52]. In addition, compared with wild-type mice, DAT-KO mice exhibit attenuated habituation [43, 53–54], which is consistent with our findings (Fig 2A). However, similar phenotypes may be generated when tyrosine hydroxylase mRNA is upregulated because both events may result in hyperactivation of D2 receptor-mediated signaling pathways followed by counterbalancing desensitization of functional D2 receptors [55].

Another finding opposite to the typical stressed model was that CHX-treated rats displayed antidepressant-like behavior in the forced swimming test. Reduced immobility of rodents is a validated experimental index of a potential antidepressant-like effect [56], and it is directly controlled, at least in part, by mesoaccumbal DAergic projections [57], although some research suggests that immobility in the forced swimming test is a readily learned adaptation to stress that conserves energy opposed to an impairment of coping with the stress [58]. The DAT hypothesis described previously may also be applicable because DAT-KO animals exhibit increased mobility than wild-type animals in the forced swimming test [43, 59].

Because we sought to evaluate the interactions between numerous behavioral variables and the effects of CHX on these variables, we employed multivariate analyses using PCA. These analyses revealed functional relationships among behavioral variables consolidated in each PC as well as the specific effects of repeated CHX administration on PC II, which included instrumental performance under the devalued condition, open-field activities before and after acute cocaine administration, and the immobility ratio of the forced swimming test (Table 1, Fig 4, and S6 Fig). As described previously, in the outcome devaluation test following the instrumental learning, a reduction in lever pressing during the devalued condition reflects the action controlled predominantly by goal-directed process that is sensitive to the current value of the outcome, whereas the outcome insensitivity manifests the predominance of habitual process to

goal-directed process. Thus, the lower PC II score of CHX-treated animals versus controls indicates that the former are prone to habitual performance. Then, a raised question is whether outcome devaluation is related to striatal DA tone, as proposed for other variables in this component. A study using DAT KO mice illustrated that the outcome devaluation effect is unaltered [60]. However, because the DAT expression in this transgenic line was inhibited at all times, other neural mechanisms may compensate for the lack of DAT function, at least in part [61]. In addition, late-developing long-term neuroadaptation induced by repeated exposure to CHX, as noted for repeated psychostimulant administration [62], could be responsible for unknown mechanism.

Although the detailed mechanism by which each PC is functionally segregated or how CHX specifically influences PC II remains unknown, striatal DA-mediated regulation may play a pivotal role in integrating most of the behavioral variables of PC II and making them sensitive to repeated CHX administration. Supporting this idea, repeated administration of methamphetamine, which induces much stronger oxidative stress than cocaine, reduces the number of DAT molecules in the rat striatum [63]. In addition, methamphetamine promotes habit formation in rats [64]. It is possible that repeated CHX administration works on DAT in a similar manner as methamphetamine. Nevertheless, the effect of CHX on neural substrates other than the DA system is also possible, and the possibility that CHX affects molecules other than glutathione must be fairly considered.

At this point, it is not conclusive that how much the alterations we observed in the present work were due to the direct effect of oxidative stress. However, because the effects of repeated CHX administration were observed when there was no change in the levels of glutathione (S1 Fig) or apoptosis (S2 Fig), it is possible that these effects were developed at a later stage as long-lasting neuroadaptations of the redox system and not by oxidative stress itself. In accordance with this hypothesis, at present, the concept that oxidative stress always acts invasively whereas the redox system always works protectively in mitigating disease is under revision [65]. Therefore, investigating the effect of repeated CHX administration on the striatal DA system and decision-making will be more important in the future. In addition, studies are required to answer the question of whether CHX-induced alterations are restored by manipulating the redox system.

In this study, we were unable to detect an effect of CHX on the performance of response discrimination, its reversal learning, or any significant group difference in PCs that were significantly loaded by these variables. In contrast, it is proposed that oxidative stress may affect reversal learning, as reported in a recent study of transgenic mice expressing a putative dominant-negative disrupted in schizophrenia 1 (DISC1). This report implicates that oxidative stress may be involved in the loss of motivation in instrumental performance with a PR schedule, accelerated habit formation of instrumental learning, and degradation of a reversal learning performance [66]. Another model exhibiting some discrepancy with our findings is glutathione-cysteine-ligase modifier (GCLM) KO mice in which glutathione synthesis is reduced. Whereas the mice were less immobile in the forced swimming tests similar to our findings, psychostimulants induced an excessive hyperlocomotion in the KO mice [13]. However, it is noteworthy that these studies employed transgenic mice with compromised DICS1 or GCLM function from the early neonatal stage but not from adulthood, and the level of glutathione reduction was far greater than that observed in our model, particularly in GCLM-KO mice. Those factors could explain the differences between these reports and our findings.

Another point to be mentioned is the difference of our findings from those in animal models of depression. Although there are some studies reporting the induction of oxidative stress in rodents exposed to chronic mild stress [67–68], Group CHX displayed a mixture of pro-depression- (attenuated habituation in novel environment and habit-prone in decision making) and antidepression-like phenotypes (blunted immobility in forced swimming and attenuated

response to acute cocaine). This could be attributed to the systemic administration of CHX, which may affect the entire brain, whereas in major depression, oxidative stress may be induced in more specific brain regions or circuits.

In conclusion, repeated CHX administration produced a habit-prone phenotype by prolonging the effect of CHX challenge on outcome devaluation, although it did not affect the acquisition or motivation of an instrumental learning trained under a PR schedule. Response discrimination and reversal learning were not affected by CHX. Repeated CHX administration reduced immobility in the forced swimming tests and blunted cocaine-induced behaviors. Combined with the results of multivariate analyses, these findings suggest that repeated CHX administration to adult rats did not generate an animal model of a certain specific mental disorder, but it may have persistently altered a group of functionally related behavioral and cognitive variables.

## Supporting Information

**S1 Fig. Dose-, timing-, and brain region-dependent effect of CHX on total glutathione levels in the nucleus accumbens (NAc) and dorsal striatum (dSt).** (A) Effects of various doses of CHX on total glutathione levels in the NAc at 1 h after acute CHX administration. Data are represented as relative ratios of the concentration of total glutathione after an acute administration of varying doses of CHX (12.5, 25, and 50 mg/kg, i.p.) to that of saline (N = 4–5 each: $^{*}p < 0.05$ vs. saline). (B) Effects of an excess amount of the glutathione precursor *N*-acetylcysteine on total glutathione levels in the NAc when co-administrated with CHX. *N*-acetylcysteine (1 g/kg) was i.p. administrated 1.5 h before acute injection of 25 mg/kg CHX (N = 4–5: $^{*}p < 0.05$, vs. saline). (C-D) Temporal effects of an acute CHX administration (25 mg/kg) on the total glutathione levels in the NAc (C; N = 4–5) and dSt (D; N = 5). Data are represented as the relative ratios of the concentrations of total glutathione at 1 and 3 h after the acute administration of 25 mg/kg of CHX to that at 1 h after acute administration of saline ($^{*}p < 0.05$, vs. saline). (E–F) Effects of repeated CHX administration for 7 days on total glutathione levels in the NAc and dSt at 1 day (E; N = 5 each; dSt, $^{*}p < 0.05$, vs. saline) or 3 weeks (F; N = 8 each) after the final CHX administration. Data are represented as relative ratios compared with the concentration of total glutathione after repeated saline administration for 7 days in the same region of the brain. Error bars represent SEM.
(PDF)

**S2 Fig. TUNEL assay of the NAc and dSt of the animals which received 25 mg/kg of CHX for 7 consecutive days.** Animals were perfused on the day following the last Vehicle/CHX administration. The brain sections from the core and shell subregions of the NAc and the dSt were compared with sections of the dSt where kainic acid was microinjected. KA: kainic acid, Veh: vehicle. Green: TUNEL, Blue: DAPI.
(TIFF)

**S3 Fig. Repeated CHX administration did not affect performance in response discrimination learning and its reversal.** (A) Mean number of trials to reach the criterion of the response discrimination training. A one-way ANOVA (Group: vehicle vs. CHX) did not reveal any significant group difference ($F[1, 16] = 1.67$). (B) Mean number of errors committed during the response discrimination training without a significant group difference ($F[1, 16] = 1.58$). (C) Mean number of trials to reach the criterion of the response reversal learning without a significant group difference ($F[1, 16] < 1$). (D) Mean numbers of the two error subtypes, perseverative and regressive, recorded during the response reversal learning. A two-way ANOVA, 2 (Group: vehicle vs. CHX) × 2 (Error type: perseverative vs. regressive), did not demonstrate

significant main effects of Group and Error type ($Fs[1, 16] < 1$) or interaction between factors ($F[1, 16] = 2.10$). Data represent the mean + SEM.
(TIF)

**S4 Fig. Repeated CHX administration did not affect spontaneous activity in a novel environment.** The spontaneous activities in actimeter, locomotor activity (A), rearing counts (B), and stereotypic counts (C) were monitored on the preceding day of the acute cocaine administration (apparatus habituation training, 25 days after repeated vehicle/CHX administration). Separate two-way ANOVAs, 2 (Group) × 12 (Time bin), revealed only significant main effects of Time bin ($F[11, 176] = 79.77$, 121.16, and 43.87; $ps < 0.001$) for locomotor activity, rearing, and stereotypy, respectively, without the Group effects (main effect or interactions: $Fs < 1.72$). Data represent the mean ± SEM.
(TIF)

**S5 Fig. Repeated CHX administration did not affect body weights.** Robust group differences were not detected during repeated CHX administration (7 days), food deprivation, or subsequent *ad-libitum* feeding. Analysis using two-way ANOVA of 2 (Group) × 7 (Time point) revealed only a significant main effect of Time point, $F(6, 96) = 1560.48$, $p < 0.001$. Data represent the mean ± SEM.
(TIF)

**S6 Fig. Structure of principal component (PC) II.** PC II accounted for 20.5% of the variance. This component was negatively loaded by the response rate in the devalued condition of devaluation test 1 as well as open field activity before acute cocaine injection, and positively loaded by open field activity after acute cocaine injection as well as the immobility ratio of the forced swimming test. Analysis of the PC scores calculated for individual animals in Groups Veh and CHX revealed that the scores of animals in Group CHX were significantly lower on PC II than those of animals in Group Veh, $t(16) = 2.96$, $p < 0.05$.
(TIF)

**S1 Table. Intercorrelations (Pearson correlation coefficient) between behavioral phenotypes.**
(DOCX)

**S2 Table. Principal component scores calculated for each animal in Groups Veh and CHX.**
(DOCX)

**S1 Text. Supporting materials and methods.**
(DOCX)

## Author Contributions

Conceived and designed the experiments: YI YM ST. Performed the experiments: YI ZL SK HN ST. Analyzed the data: YI ST. Contributed reagents/materials/analysis tools: YI ZL SK HN ST. Wrote the paper: YI YM ST.

## References


1. Karatsoreos IN, McEwen BS (2011) Psychological allostasis: resistance, resilience and vulnerability. Trends Cogn Sci 15:576–584. doi: 10.1016/j.tics.2011.10.005 PMID: 22078931
2. Suo L, Zhao L, Si J, Liu J, Zhu W, et al. (2013) Predictable chronic mild stress in adolescence increases resilience in adulthood. Neuropsychopharmacology 38:1387–1400. doi: 10.1038/npp.2013.67 PMID: 23478858

3. Arnsten AF (2009) Stress signaling pathways that impair prefrontal cortex structure and function. Nat Rev Neurosci 10:410–422. doi: 10.1038/nrn2648 PMID: 19455173
4. Feder A, Nestler EJ, Charney DS (2009) Psychobiology and molecular genetics of resilience. Nat Rev Neurosci 10:446–457. doi: 10.1038/nrn2649 PMID: 19455174
5. Koob GF (2008) A role for brain stress systems in addiction. Neuron 59: 11–34. doi: 10.1016/j.neuron.2008.06.012 PMID: 18614026
6. Colaianna M, Schiavone S, Zotti M, Tucci P, Morgese MG, et al. (2013) Neuroendocrine profile in a rat model of psychosocial stress: relation to oxidative stress. Antioxid Redox Signal 18: 1385–1399. doi: 10.1089/ars.2012.4569 PMID: 23320850
7. Nikolaidis MG, Kyparos A, Spanou C, Paschalis V, Theodrou AA, et al. (2012) Redox biology of exercise: an integrative and comparative consideration of some overlooked issues. J Exp Biol 215:1615–1625. doi: 10.1242/jeb.067470 PMID: 22539728
8. Patki G, Solanki N, Atooz F, Allam F, Salim S (2013) Depression, anxiety-like behavior and memory impairment are associated with increased oxidative stress and inflammation in a rat model of social defeat. Brain Res 1539:73–86. doi: 10.1016/j.brainres.2013.09.033 PMID: 24096214
9. Schiavone S, Jaquet V, Trabace L, Krause K-H (2012) Severe life stress and oxidative stress in the brain: from animal models to human pathology. Antioxid Redox Signal 18:1475–1490. doi: 10.1089/ars.2012.4720 PMID: 22746161
10. Do KQ, Cabungcal JH, Frank A, Steullet P, Cuenod M (2009) Redox dysregulation, neurodevelopment, and schizophrenia. Curr Opin Neurobiol 19:220–230. doi: 10.1016/j.conb.2009.05.001 PMID: 19481443
11. Emiliani FE, Sedlak TW, Sawa A (2014) Oxidative stress and schizophrenia: recent breakthroughs from an old story. Curr Opin Psychiatry 27:185–190. doi: 10.1097/YCO.0000000000000054 PMID: 24613987
12. Yang Y, Dieter MZ, Chen Y, Shertzer HG, Nebert DW, et al. (2002) Initial characterization of the glutamate-cysteine ligase modifier subunit Gclm (-/-) knockout mouse: novel model system for a severely compromised oxidative stress response. J Biol Chem 277:49446–49452. PMID: 12384496
13. Kulak A, Cuenod M, Do KQ (2012) Behavioral phenotyping of glutathione-deficit mice: relevance to schizophrenia and bipolar disorder. Behav Brain Res 226:563–570. doi: 10.1016/j.bbr.2011.10.020 PMID: 22033334
14. Dringen R (2000) Metabolism and functions of glutathione in brain. Prog Neurobiol 62:649–671. PMID: 10880854
15. Marukawa T, Sai M, Tochino Y (1989) Methods for depleting brain glutathione. Life Sci 44:417–424. PMID: 2918812
16. Cruz-Aguado R, Almaguer-Melian W, Diaz CM, Lorigados L, Bergado J (2001) Behavioral and biochemical effects of glutathione depletion in the rat brain. Brain Res Bull 55: 327–33. PMID: 11489339
17. Wilson CB, McLaughlin LD, Nair A, Ebenezer PJ, Dange R, et al. (2013) Inflammation and oxidative stress are elevated in the brain, blood, and adrenal glands during the progression of post-traumatic stress disorder in a predator exposure animal model. PLoS One 8(10): e76146. doi: 10.1371/journal.pone.0076146 PMID: 24130763
18. Doya K (2008) Modulators of decision making. Nat Neurosci 11:410–416. doi: 10.1038/nn2077 PMID: 18368048
19. Maia TV, Frank MJ (2011) From reinforcement learning models to psychiatric and neurological disorders. Nat Neurosci 14: 154–162. doi: 10.1038/nn.2723 PMID: 21270784
20. Lammel S, Lim BK, Malenka RC (2014) Reward and aversion in a heterogeneous midbrain dopamine system. Neuropharmacology 76 PtB: 351–359. doi: 10.1016/j.neuropharm.2013.03.019 PMID: 23578393
21. Simeone A, Di Salvio M, Di Giovannantonio LG, Acampora D, Omodei D, et al (2011) The role of otx2 in adult mesencephalic-diencephalic dopaminergic neurons. Mol Neurobiol 43: 107–113. doi: 10.1007/s12035-010-8148-y PMID: 21086067
22. Roberts DS, Richardson N (1992) Self-administration of psychomotor stimulants using progressive ratio schedules of reinforcement. In: Boulton A, Baker G, Wu P, editors. Animal Models of Drug Addiction: Humana Press. pp. 233–269.
23. Overstreet D (2012) Modeling depression in animal models. In: Kobeissy FH, editor. Psychiatric Disorders: Humana Press. pp. 125–144.
24. R Core Team (2012) R: A language and environment for statistical computing.
25. De Bundel D, Schallier A, Loyens E, Fernando R, Miyashita H, et al. (2011) Loss of system x(c)- dose not induce oxidative stress but decreases extracellular glutamate in hippocampus and influences

spatial working memory and limbic seizure susceptibility. J Neurosci 31: 5792–5803. doi: 10.1523/JNEUROSCI.5465-10.2011 PMID: 21490221

26. Rongzhu L, Suhua W, Guangwei X, Chinlan R, Fangan H, et al. (2009) Effects of acrylonitrile on antioxidant status of different brain regions in rats. Neurochem Int 55: 552–557. doi: 10.1016/j.neuint.2009.05.009 PMID: 19463879
27. Dringen R, Pfeiffer B, Hamprecht B (1999) Synthesis of the antioxidant glutathione in neurons: supply by astrocytes of CysGly as precursor for neuronal glutathione. J Neurosci 19:562–569. PMID: 9880576
28. Randall PA, Pardo M, Nunes EJ, Lopez Cruz L, Vemuri VK, et al (2012) Dopaminergic modulation of effort-related choice behavior as assessed by a progressive ratio chow feeding choice task: pharmacological studies and the role of individual differences. PLoS One 7: e47934. doi: 10.1371/journal.pone.0047934 PMID: 23110135
29. Dickinson A (1985) Actions and habits: the development of behavioural autonomy. Philos Trans R Soc B 308: 67–78.
30. Graybiel AM (2008) Habits, rituals, and the evaluative brain. Annu Rev Neurosci 31: 359–387. doi: 10.1146/annurev.neuro.29.051605.112851 PMID: 18558860
31. Hilario MR, Costa RM (2008) High on habits. Front Neurosci 2: 208–217. doi: 10.3389/neuro.01.030.2008 PMID: 19225594
32. Holland PC (2004) Relations between Pavlovian-instrumental transfer and reinforcer devaluation. J Exp Psychol Anim Behav Process 30: 104–117. PMID: 15078120
33. Yin HH, Knowlton BJ (2006) The role of the basal ganglia in habit formation. Nat Rev Neurosci 7: 464–476. PMID: 16715055
34. Shukitt-Hale B, Erat SA, Joseph JA (1998) Spatial learning and memory deficits induced by dopamine administration with decreased glutathione. Free Radical Biol Med 24: 1149–1158. PMID: 9626569
35. Choy KH, Dean O, Berk M, Bush AI, van den Buuse M (2010) Effects of N-acetyl-cysteine treatment on glutathione depletion and a short-term spatial memory deficit in 2-cyclohexene-1-one-treated rats. Eur J Pharmacol 649: 224–228. doi: 10.1016/j.ejphar.2010.09.035 PMID: 20868666
36. Dean O, Bush AI, Berk M, Copolov DL, van den Buuse M (2009) Glutathione depletion in the brain disrupts short-term spatial memory in the Y-maze in rats and mice. Behav Brain Res 198: 258–262. doi: 10.1016/j.bbr.2008.11.017 PMID: 19061918
37. Castagne V, Cuenod M, Do KQ (2004) An animal model with relevance to schizophrenia: sex-dependent cognitive deficits in osteogenic disorder-Shionogi rats induced by glutathione synthesis and dopamine uptake inhibition during development. Neuroscience 123: 821–834. PMID: 14751276
38. Schwabe L, Wolf OT (2010) Socially evaluated cold pressor stress after instrumental learning favors habits over goal-directed action. Psychoneuroendocrinology 35: 977–986. doi: 10.1016/j.psyneuen.2009.12.010 PMID: 20071096
39. Schwabe L, Wolf OT (2009) Stress prompts habit behavior in humans. J Neurosci 29: 7191–7198. doi: 10.1523/JNEUROSCI.0979-09.2009 PMID: 19494141
40. Schwabe L, Wolf OT (2011) Stress-induced modulation of instrumental behavior: from goal-directed to habitual control of action. Behav Brain Res 219: 321–328. doi: 10.1016/j.bbr.2010.12.038 PMID: 21219935
41. Prasad BM, Sorg BA, Ulibarri C, Kalivas PW (1995) Sensitization to stress and psychostimulants. Involvement of dopamine transmission versus the HPA axis. Ann N Y Acad Sci. 771:617–625. PMID: 8597435
42. Groppetti A, Zambotti F, Biazzi A, Mantegazza P (1974) Amphetamine and cocaine on amine turnover. Biochem Pharmacol 23, Supplement 1: 756–764.
43. Giros B, Jaber M, Jones SR, Wightman RM, Caron MG (1996) Hyperlocomotion and indifference to cocaine and amphetamine in mice lacking the dopamine transporter. Nature 379: 606–612. PMID: 8628395
44. Thomsen M, Han DD, Gu HH, Caine SB (2009) Lack of cocaine self-administration in mice expressing a cocaine-insensitive dopamine transporter. J Pharmacol Exp Ther 331: 204–211. doi: 10.1124/jpet.109.156265 PMID: 19602552
45. Kelly PH, Iversen SD (1976) Selective 6OHDA-induced destruction of mesolimbic dopamine neurons: abolition of psychostimulant-induced locomotor activity in rats. Eur J Pharmacol 40: 45–56. PMID: 1033072
46. Kelly PH, Seviour PW, Iversen SD (1975) Amphetamine and apomorphine responses in the rat following 6-OHDA lesions of the nucleus accumbens septi and corpus striatum. Brain Res 94: 507–522. PMID: 1171714

47. Pijnenburg AJ, Honig WM, Van Rossum JM (1975) Inhibition of d-amphetamine-induced locomotor activity by injection of haloperidol into the nucleus accumbens of the rat. Psychopharmacologia 41: 87–95. PMID: 1098086

48. Asher IM, Aghajanian GK (1974) 6-Hydroxydopamine lesions of olfactory tubercles and caudate nuclei: effect on amphetamine-induced stereotyped behavior in rats. Brain Res 82: 1–12. PMID: 4373138

49. Creese I, Iversen SD (1975) The pharmacological and anatomical substrates of the amphetamine response in the rat. Brain Res 83: 419–436. PMID: 234270

50. Garcia-Fuster MJ, Perez JA, Clinton SM, Watson SJ, Akil H (2010) Impact of cocaine on adult hippocampal neurogenesis in an animal model of differential propensity to drug abuse. Eur J Neurosci 31: 79–89. PMID: 20104651

51. Szumlinski KK, Frys KA, Kalivas PW (2003) Pretreatment with serotonin 5-HT(3) receptor antagonists produces no observable blockade of long-term motor sensitization to cocaine in rats. Psychopharmacology (Berl) 165: 329–336. PMID: 12451437

52. Sora I, Wichems C, Takahashi N, Li XF, Zeng Z, et al. (1998) Cocaine reward models: conditioned place preference can be established in dopamine- and in serotonin-transporter knockout mice. Proc Natl Acad Sci U S A 95: 7699–7704. PMID: 9636213

53. Spielewoy C, Roubert C, Hamon M, Nosten-Bertrand M, Betancur C, et al. (2000) Behavioural disturbances associated with hyperdopaminergia in dopamine-transporter knockout mice. Behav Pharmacol 11: 279–290. PMID: 11103882

54. Zhuang X, Oosting RS, Jones SR, Gainetdinov RR, Miller GW, et al. (2001) Hyperactivity and impaired response habituation in hyperdopaminergic mice. Proc Natl Acad Sci U S A 98: 1982–1987. PMID: 11172062

55. Beaulieu JM, Gainetdinov RP (2011) The physiology, signaling, and pharmacology of dopamine receptors. Pharmacol Rev 63: 182–217. doi: 10.1124/pr.110.002642 PMID: 21303898

56. Porsolt RD, Le Pichon M, Jalfre M (1977) Depression: a new animal model sensitive to antidepressant treatments. Nature 266: 730–732. PMID: 559941

57. Tye KM, Mirzabekov JJ, Warden MR, Ferenczi EA, Tsai HC, et al. (2013) Dopamine neurons modulate neural encoding and expression of depression-related behaviour. Nature 493: 537–541. doi: 10.1038/nature11740 PMID: 23235822

58. West AP (1990) Neurobehavioral studies of forced swimming: the role of learning and memory in the forced swim test. Prog Neuropsychopharmacol Biol Psychiatry 14: 863–877. PMID: 2277851

59. Perona MT, Waters S, Hall FS, Sora I, Lesch KP, et al. (2008) Animal models of depression in dopamine, serotonin, and norepinephrine transporter knockout mice: prominent effects of dopamine transporter deletions. Behav Pharmacol 19: 566–574. doi: 10.1097/FBP.0b013e32830cd80f PMID: 18690111

60. Yin HH, Zhuang X, Balleine BW (2006) Instrumental learning in hyperdopaminergic mice. Neurobiol Learn Mem 85: 283–288. PMID: 16423542

61. Ramadan E, Chang L, Chen M, Ma K, Hall FS, et al. (2012) Knocking out the dopamine reuptake transporter (DAT) does not change the baseline brain arachidonic acid signal in the mouse. Int J Neurosci 122: 373–380. doi: 10.3109/00207454.2012.665972 PMID: 22376027

62. Kalivas PW, Toda S, Bowers MS, Baker DA, Ghasemzadeh MB (2003). The temporal sequence of change in gene expression by drugs of abuse. Methods Mol Med 79: 3–11. PMID: 12506686

63. Hashimoto K, Tsukada H, Nishiyama S, Fukumoto D, Kakiuchi T, et al. (2004) Protective effects of N-acetyl-L-cysteine on the reduction of dopamine transporters in the striatum of monkeys treated with methamphetamine. Neurosoychopharmacology 29: 2018–2023. PMID: 15199373

64. Nelson A, Killcross S (2006) Amphetamine exposure enhances habit formation. J Neurosci 26: 3805–3812. PMID: 16597734

65. Sasaoka T (2012) Oxidative stress in neurodegenerative diseases: friends and foe. Clin Exp Pharmacol Physiol: 39: 597–98. doi: 10.1111/j.1440-1681.2012.05723.x PMID: 22738365

66. Johnson AW, Jaaro-Peled H, Shahani N, Sedlak TW, Zoubovsky S, et al. (2013) Cognitive and motivational deficits together with prefrontal oxidative stress in a mouse model for neuropsychiatric illness. Proc Natl Acad Sci U S A 110:12462–12467. doi: 10.1073/pnas.1307925110 PMID: 23840059

67. Lucca G, Comim CM, Valvassori SS, Reus GZ, Vuolo F, et al. (2009) Effects of chronic mild stress on the oxidative parameters in the rat brain. Neurochem Int 54:358–362. doi: 10.1016/j.neuint.2009.01.001 PMID: 19171172

68. Tagliari T, dos Santos TM, Cunha AA, Lima DD, Delwing D, et al. (2010) Chronic variable stress induces oxidative stress and decreases butyrylcholinesterase activity in blood of rats. J Neural Transm 117:1067–1076. doi: 10.1007/s00702-010-0445-0 PMID: 20686907
